# NOX4 is a potential therapeutic target in septic acute kidney injury by inhibiting mitochondrial dysfunction and inflammation

**DOI:** 10.7150/thno.81240

**Published:** 2023-05-08

**Authors:** Jiameng Li, Liya Wang, Bo Wang, Zhuyun Zhang, Luojia Jiang, Zheng Qin, Yuliang Zhao, Baihai Su

**Affiliations:** 1 Center of Gerontology and Geriatrics, West China Hospital, Sichuan University, Chengdu 610041, China.; 2 Department of Nephrology, Kidney Research Institute, West China Hospital, Sichuan University, Chengdu 610041, China.; 3 Department of Nephrology, Jiujiang No. 1 People's Hospital, Jiujiang 332000, China.

**Keywords:** Septic AKI, NOX4, mitochondrial dysfunction, inflammation, apoptosis

## Abstract

**Rationale:** Sepsis is a severe clinical syndrome featured through organ dysfunction due to infection, while the accompanying acute kidney injury (AKI) is linked to significant incidence of morbidity as well as mortality. Recently, emerging evidence has revealed that nicotinamide adenine dinucleotide phosphate (NADPH) oxidase 4 (NOX4) is implicated in various renal diseases, while its role and modulation in septic acute kidney injury (S-AKI) remains largely unknown.

**Methods:** *In vivo*, S-AKI in wild-type and renal tubular epithelial cell (RTEC)-specific NOX4 knockout mice was induced by lipopolysaccharides (LPS) injection or cecal ligation and puncture (CLP). *In vitro*, TCMK-1 (mouse kidney tubular epithelium cell line) cells were treated with LPS. Serum and supernatant biochemical, mitochondrial dysfunctional, inflammatory and apoptotic parameters were measured and compared across groups. The activation of reactive oxygen species (ROS) and NF-κB signaling was also assessed.

**Results:** NOX4 was predominantly upregulated in RTECs of S-AKI mouse model induced by LPS/CLP and cultured TCMK-1 cells exposed to LPS. RTEC-specific deletion of NOX4 or pharmacological inhibition of NOX4 by GKT137831 both alleviated LPS/CLP-injured renal function and pathology in mice. Furthermore, NOX4 inhibition alleviated mitochondrial dysfunction supported by ultrastructural damage, reduction of ATP production and mitochondrial dynamics imbalance, together with inflammation and apoptosis in kidney injured by LPS/CLP and TCMK-1 cells injured by LPS, while NOX4 overexpression aggravated the above-mentioned indices in TCMK-1 cells with LPS stimulation. Mechanism-wise, the raised NOX4 in RTECs may induce ROS and NF-κB signaling activation in S-AKI.

**Conclusions:** Collectively, genetic or pharmacological inhibition of NOX4 protects from S-AKI by reducing generation of ROS and activation of NF-κB signal, which suppress mitochondrial dysfunction, inflammation together with apoptosis. NOX4 may act as a novel target for the S-AKI therapy.

## Introduction

Sepsis, characterized by systemic inflammation, host immune disorders as well as multi-organ dysfunction, accounts for approximately 50% acute kidney injury (AKI) amongst critical care patients 1, 2. Unfortunately, septic acute kidney injury (S-AKI) has been highly associated to poor clinical outcome. In critical patients suffering from AKI, S-AKI is associated with greater in-hospital mortality risk and greater length of stay compared to other causes of AKI 3, 4. In spite of this, the underlying pathogenesis of S-AKI remains poorly understood, resulting in nonspecific and reactive treatment 4, 5.

Nicotinamide adenine dinucleotide phosphate (NADPH) oxidase 4 (NOX4) is a constitutive enzyme expressed in the kidney and primarily contributes to the growth in reactive oxygen species (ROS) 6, and exerts a pivotal part in the modulation of oxidative stress and downstream signals 7-10. Growing evidence showed that NOX4 plays a role in the pathogenesis of various kidney diseases, such as diabetic nephropathy 11, hypertensive nephropathy 12, obstructive nephropathy 13, as well as AKI induced by ischemia/reperfusion injury and cisplatin 14-18. Recent research suggested NOX4 was regulated by SH3YL1 in generating ROS in LPS-induced AKI model 19. Our previous study demonstrated that over-expression of NOX4 in renal tubular epithelial cells (RTECs) in LPS-induced AKI was ameliorated after Maresin 1 administration 20, while the therapeutic potential and regulatory mechanism of targeting NOX4 in SAKI remain largely unknown.

In the current work, we demonstrated that NOX4 is induced by LPS/CLP *in vivo* and LPS *in vitro*. NOX4 gene knockout or silencing, and NOX4 inhibition by GKT137831 all significantly ameliorated S-AKI, while NOX4 overexpression exacerbated kidney tubular epithelial cell injury induced by LPS. Mechanistically, NOX4 may mediates the ROS/NF-κB signal activation to stimulate mitochondrial dysfunction, inflammation as well as apoptosis. Our findings displayed that NOX4 may be a prospective therapeutic target for S-AKI.

## Materials and Methods

### Reagents and Antibodies

GKT137831 (S7171) was provided by Selleck (Shanghai, China). LPS (L8880, *E. coli* 055:B5) was acquired from Solarbio (Beijing, China). NOX4 siRNA (siNOX4) and negative control (NC) siRNA (siNC) were obtained from RiboBio (Guangzhou, China). Adenoviruses expressing NOX4 (Ad-NOX4) or no NOX4 (Ad-Null) were provided by Hanbio Tech (Shanghai, China) at titers of 1 × 10^10^ PFU/mL. The primary antibodies that utilized in the study are presented in Table S1.

### Animal Experiments

Experimental procedures for animals were endorsed by the Institutional Animal Care and Use Committee, West China Hospital, Sichuan University (Approval No. 2018168A). Male C57BL/6J mice (aged between 6-8 weeks, with a weight of 18-25 g) and RTEC-specific NOX4 knockout (Cdh16-Cre^+^ NOX4^fl/fl^, NOX4^tecKO^) mice were intraperitoneally injected with LPS or subjected to cecal ligation and puncture (CLP) to establish the model of S-AKI. All mice were randomly divided into groups with 5 mice in each group. With an automatic biochemical analyzer (Chemray 240, Rayto Life and Analytical Sciences, Shenzhen, China), the serum creatinine (Scr) and blood urea nitrogen (BUN) were identified. The AKI mouse model was created successfully when the levels of Scr rose to twice the levels of their control littermates. The details of RTEC-specific NOX4 knockout mice generation as well as animal experiments are presented in the Supplementary Methods.

### Cell Culture and Treatments of TCMK-1

Mouse kidney tubular epithelium cells (TCMK-1, ATCC^®^ CCL-139^TM^) purchased from American Type Culture Collection (Manassas, VA, USA) were cultivated in MEM/EBSS medium (Gibco, Rockville, MD, USA) that was supplemented with 10% fetal bovine serum (FBS) at 37 °C under a humidified atmosphere of 95% air and 5% CO_2_. For LPS treatment, TCMK-1 cells (50-60% confluent) were starved in MEM/EBSS medium containing 0.5% FBS for 24 h, followed by exposure to LPS 100 μg/ml for 12 h. GKT137831 10 μM was applied to pretreat TCMK-1 cells at 30 min before LPS administration. The details of transfection of siNC, siNOX4, Ad-Null and Ad-NOX4 in TCMK-1 cells are presented in the Supplementary Methods.

### Immunofluorescence Staining

Kidney specimens were sliced into sections (4 μm) on a cryostat. The TCMK-1 cell slides were fixed with 4% paraformaldehyde. PBS with 5% bovine serum was applied to block the non-specific binding sites for 30 min at 37 °C. The kidney sections and cell slides were subsequently labeled with appropriate primary antibodies overnight at 4 °C. Following washing with PBS, the appropriate secondary antibodies were applied for 1 h. The cell nuclei were counter-stained with DAPI (Servicebio, Wuhan, China). Images were observed and next captured by an AxioCamHRc digital camera (Carl Zeiss, Jena, Germany) with ZEN 2012 microscopy software (blue edition).

### Immunohistochemistry

Tissue sections were dewaxed, dehydrated and then washed by PBS. After removing endogenous peroxidase with 3% H_2_O_2_, antigen retrieval was performed by applying citrate. The primary antibody anti-NOX4 (1:100) was subjected to overnight incubation at 4 °C. Following PBS washings, biotinylated secondary antibody was used to incubate the slices at room temperature for 30 min. Images of random renal cortex sections were observed and captured by an AxioCamHRc digital camera (Carl Zeiss, Jena, Germany) with ZEN 2012 microscopy software (blue edition) at a magnification of ×200.

### Histological Staining and Evaluation

Kidney tissues were fixed with 4% paraformaldehyde for paraffin embedding and kidney sections were subjected to hematoxylin-eosin (HE) staining. HE-stained tissue sections were viewed with a light microscope at ×200 or ×400 magnifications. The tissue damage was scored in a blinded manner on the basis of the proportion of damaged renal tubules and histological injury indicated by brush border loss, tubular dilation/flattening, tubular degeneration, tubular cast formation as well as vacuolization. Tissue injury was scored on a scale of 0-4, with 0 to 4 respectively representing 0%, < 25%, 26‐50%, 51‐75% and > 76% of impaired renal tubules 21. For each sample, 10 fields of ×400 amplification were examined and averaged.

### TUNEL staining and Annexin V-FITC/propidium iodide assay

*In vivo*, according to the instructions, renal tissue cell apoptosis was investigated by terminal deoxynucleotidyl transferase‐mediated dUTP nick-end labeling (TUNEL) staining using the DeadEnd™ Fluorometric TUNEL System (Promega, Wisconsin, USA). Detection of positive cells was implemented with by fluorescence microscopy (Carl Zeiss, Jena, Germany) at ×200 magnification. For each sample, 10 images were randomly selected for counting to identify the amount of apoptotic cell nuclei. *In vitro*, TUNEL staining was carried out using the Fluorescein (FITC) TUNEL Cell Apoptosis Detection Kit (Servicebio, Wuhan, China). The ratio of TUNEL‐positive cells was evaluated in 3 visual fields.

The apoptosis rate of cultured TCMK-1 cells was assessed by an Annexin V-FITC/propidium iodide (PI) kit (4A Biotech, Beijing, China) in combination with flow cytometry (Beckman Coulter, Brea, CA). The preparation of cells was performed following the protocol of manufacturer. In brief, cells were washed twice with PBS and then resuspended in 1×Binding Buffer (100 μl). Afterwards, the cell suspension was added with Annexin V (5 μl) and incubated at room temperature in darkness for 15 min. Lastly, PI (5 μl) and 1× Binding Buffer (400 μl) were inserted and the cells were analyzed by flow cytometry within 1 h.

### Transmission Electron Microscopy

Fresh kidney cortex or TCMK-1 cells were prefixed with cold 3% glutaraldehyde for 2 h under a temperature of 4 °C. Afterwards, the samples were post-fixed in 1% osmium tetroxide, dehydrated in series acetone, infiltrated in Epox 812 for a longer period, and then embedded. The semithin sections were stained with methylene blue, and ultrathin sections were cut with a diamond knife and next stained with uranyl acetate and lead citrate. Sections were viewed by a Hitachi H-7650 electron microscope (kidney tissues) or a JEM-1400FLASH transmission electron microscope (TCMK-1 cells).

### Cell Viability Assay

Cell viability was identified via exploiting the Cell Counting Kit-8 assay (CCK-8, Dojindo Molecular Technologies, Gaithersburg, MD) following the manufacturer's instructions. TCMK-1 cells (5000 cells/well) were seeded in 96-well plates for 24 h at 37 °C in an environment with 5% CO_2_. After treated with LPS or GKT137831 for 12 h, the medium in each well was substituted by medium containing CCK-8 solution (10 μl) and incubated in absence of light under the same conditions for 1 h. In the end, the absorbance of solution in each well was identified at a wavelength of 450 nm using a microplate reader (Synergy Mx, Biotek, Winooski, VT, USA).

### ATP Production Assay

ATP generation in the kidney tissue was detected by an ATP Assay Kit (Beyotime Biotechnology, Shanghai, China) following the experimental protocol. Renal samples were lysed with a glass homogenizer on ice and next centrifuged at 12,000 × g for 5 min. The supernatant was collected for luminescence analysis via a multifunctional microplate reader. A bicinchoninic acid (BCA) protein assay kit (Biosharp, Hefei, China) was employed so that the proteins concentration in samples can be detected to eliminate the errors resulting from differences in protein content during sample preparation. Finally, the concentration of ATP was converted into mmol/mg protein.

### ELISA

The TNF-α, IL-6 and IL-1β levels in the serum of mice or supernatant of TCMK-1 cells were identified by utilizing Mouse TNF-α ELISA kit (Neobioscience Technology, Shenzhen, China), Mouse IL-6 Uncoated ELISA (Thermo Fisher Scientific, Vienna, Austria) along with Mouse IL-1 beta Uncoated ELISA (Thermo Fisher Scientific, Vienna, Austria) according to the directions of the manufacturer.

### Quantitative Real-time Polymerase Chain Reaction (RT-qPCR) Analysis

Total RNA was extracted from frozen kidney tissues or TCMK-1 cells using the FastPure^®^ Cell/Tissue Total RNA Isolation Kit V2 (Vazyme Biotech Co., Ltd., Nanjing, China). RT-qPCR was carried out as described before 20. The primer sequences of the detected genes are listed in Table S2. The results were calculated using the 2^-ΔΔCt^ method, and GAPDH was used as the internal reference control.

### Western Blot Analysis

The total protein extraction was implemented from frozen kidney cortex and cultured TCMK-1 cells, and western blot analysis was carried out as formerly reported 20. Protein bands was quantified with the ImageJ program (NIH, Bethesda, MD, USA) and GAPDH was applied as the internal reference control.

### Measurement of ROS and mitochondrial ROS (mtROS)

Detection of ROS in the kidney tissues was performed with fluorescence microscopy (Nikon, Tokyo, Japan) and stained *in situ* with the oxidative fluorescent dye dihydroethidium (DHE) (Sigma‒Aldrich, St. Louis, MO, USA). Nuclei were counter-stained by 4,6-diamidino-2-phenylindole (DAPI) (Servicebio, Wuhan, China). The ROS in TCMK-1 cells was measured by flow cytometry (Beckman Coulter, Brea, CA) applying 2',7'-dichlorofluorescein diacetate (DCFH-DA) according to the manufacturer's guidelines (Beyotime Biotechnology, Shanghai, China).

The mtROS level of TCMK-1 cells was detected with MitoSOX staining. Cells were incubated with MitoSOX reagent (Invitrogen, CA, USA) working solution (500 nM) for 10 min at 37 °C while protected from light. Washed cells gently three times with warm Hanks Balanced Salt Solution (HBSS), then mounted in warm HBSS. The images were acquired using a Leica SP8 confocal microscope.

### Statistical Analysis

Each experiment was repeated at least 3 times. The obtained data are expressed as the means ± standard deviations (SD) of the indicated number of separate experiments. The number of biological replicates is presented by individual data points in each bar graph. ANOVA followed by Tukey's post hoc test was employed to identify significant differences among multiple groups. Statistical analyses were performed with GraphPad Prism 9.3 (GraphPad Software, San Diego, CA, USA). Data were regarded as significant when the *P* value was lower than 0.05.

## Results

### NOX4 deficiency and GKT137831 treatment both protect renal function and protect against pathological injury in S-AKI mice

**Figure 1:**
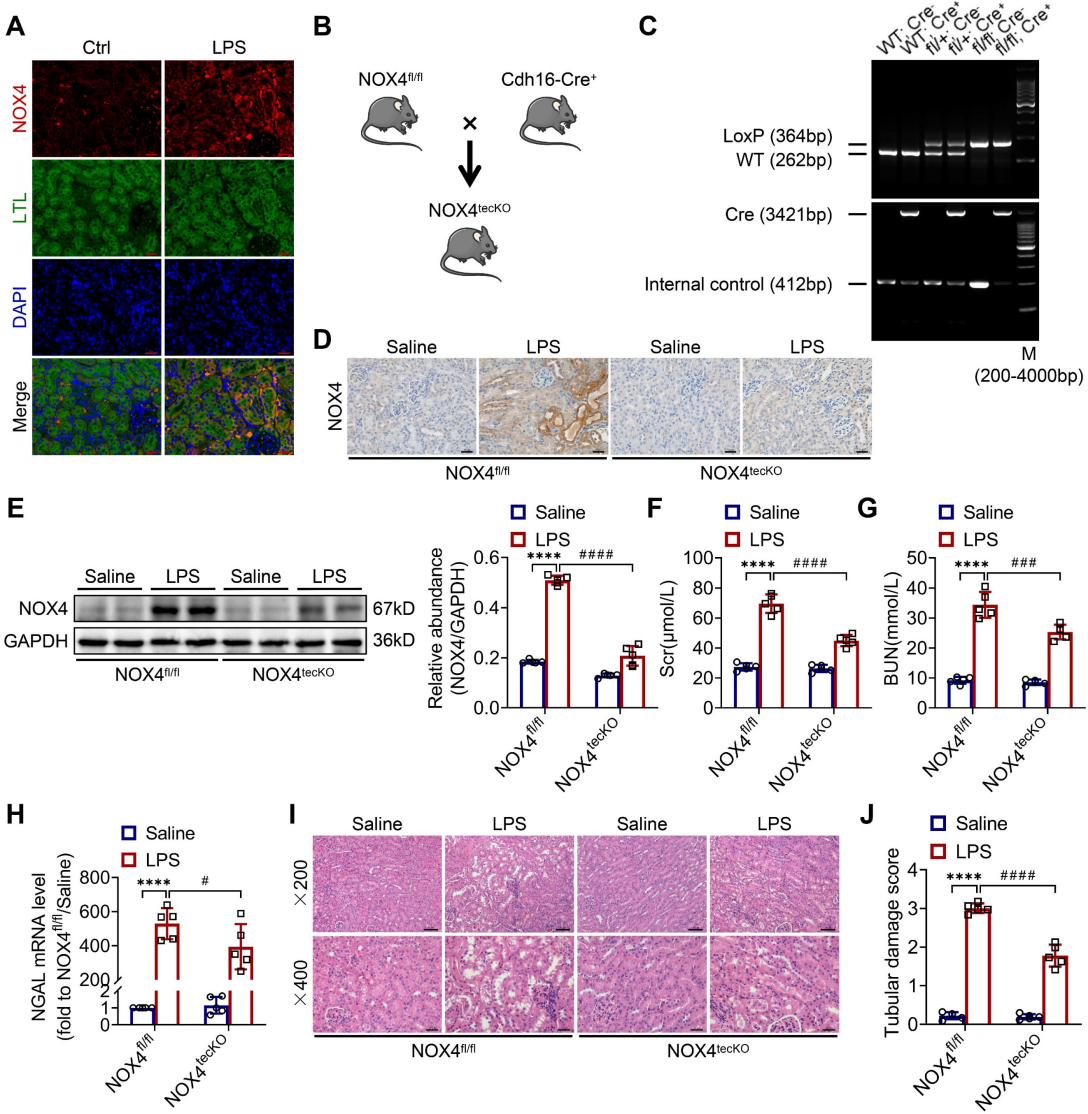
** RTEC-specific deletion of NOX4 alleviated renal injury in LPS-induced S-AKI mice.** (A) Representative micrographs of double immunofluorescence staining of NOX4 (red) and the proximal RTEC marker LTL (green) in the renal cortex (×400, scale bars = 20 μm). (B) Mating strategy to generate NOX4 conditional knockout in mouse RTECs. (C) Successful transmission of Cdh16-Cre and NOX4^fl/fl^ was confirmed by PCR genotyping. (D) Representative images of immunochemistry staining of NOX4 in kidney tissue sections (×400, scale bars = 20 μm). (E) Western blot analysis of NOX4 protein expression in the renal cortex and quantified by densitometry. (F) Scr and (G) BUN levels in different groups of mice. (H) Renal NGAL mRNA expression measured by RT‒qPCR. (I) Representative images of HE staining (×200, scale bars = 50 μm; ×400, scale bars = 20 μm) and (J) tubular damage scores of kidney tissues. Data are represented as the mean ± SD, n = 5. ^****^P < 0.0001 vs NOX4^fl/fl^/Saline; ^#^P < 0.05, ^###^P < 0.001, ^####^P < 0.0001 vs NOX4^fl/fl^/LPS.

First, we revealed that the expression of NOX4 was notably raised in proximal renal tubular epithelial cells (marked by Lectin) of S-AKI mice induced by LPS, as demonstrated by double immunofluorescence staining (Figure 1A). Next, for the evaluation of the role of RTEC-specific NOX4 *in vivo*, we built NOX4^flox/flox^ (NOX4^fl/fl^) mice by CRISPR/Cas9-stimulated homologous recombination (Figure S1). We then crossed NOX4^fl/fl^ mice with RTEC-specific cadherin-16 (Cdh16)-Cre transgenic mice to generate conditional NOX4 knockout (Cdh16-Cre^+^ NOX4^fl/fl^ here referred to as NOX4^tecKO^) mice (Figure 1B). Littermates of NOX4^fl/fl^ mice matched for age and sex were regarded as controls. The genotypes of NOX4^tecKO^ and control mice were confirmed by PCR and DNA agarose gel electrophoresis (Figure 1C). As shown in Figure 1D, NOX4 was mainly upregulated in RTECs of NOX4^fl/fl^ mice after LPS administration but inhibited in most RTECs in NOX4^tecKO^ mice. Meanwhile, in comparison to NOX4^fl/fl^ mice, the protein level of NOX4 was decreased in the kidney of NOX4^tecKO^ mice before and after S-AKI modeling (Figure 1E), suggesting that NOX4 was effectively deleted in RTECs. In addition, no apparent differences were found between NOX4^tecKO^ and NOX4^fl/fl^ mice in terms of renal function, neutrophil gelatinase-associated lipocalin (NGAL) or histopathologic injury (Figure 1F-J).

As illustrated in Figure 1F-G, Scr and BUN levels were clearly reduced in NOX4^tecKO^ mice subjected to LPS in comparison to NOX4^fl/fl^ mice. Renal mRNA expression of NGAL was also decreased in NOX4^tecKO^ mice after 12 h of LPS exposure (Figure 1H). Also, NOX4^tecKO^ mice showed effective amelioration of pathological injury characterized by cast formation, tubular dilatation, brush border loss and tubular epithelial vacuolation after LPS treatment in contrast to that of NOX4^fl/fl^ mice. (Figure 1I-J). Similarly, NOX4 expression of kidney was also induced by CLP, and then decreased in CLP-injured kidneys of NOX4^tecKO^ mice (Figure S2A). In the meantime, the kidney dysfunction (Figure S2B-C) and tubular damage (Figure S2D-F) of CLP-induced NOX4^tecKO^ mice were also markedly alleviated. These findings demonstrated that RTEC-specific deletion of NOX4 attenuated S-AKI.

**Figure 2:**
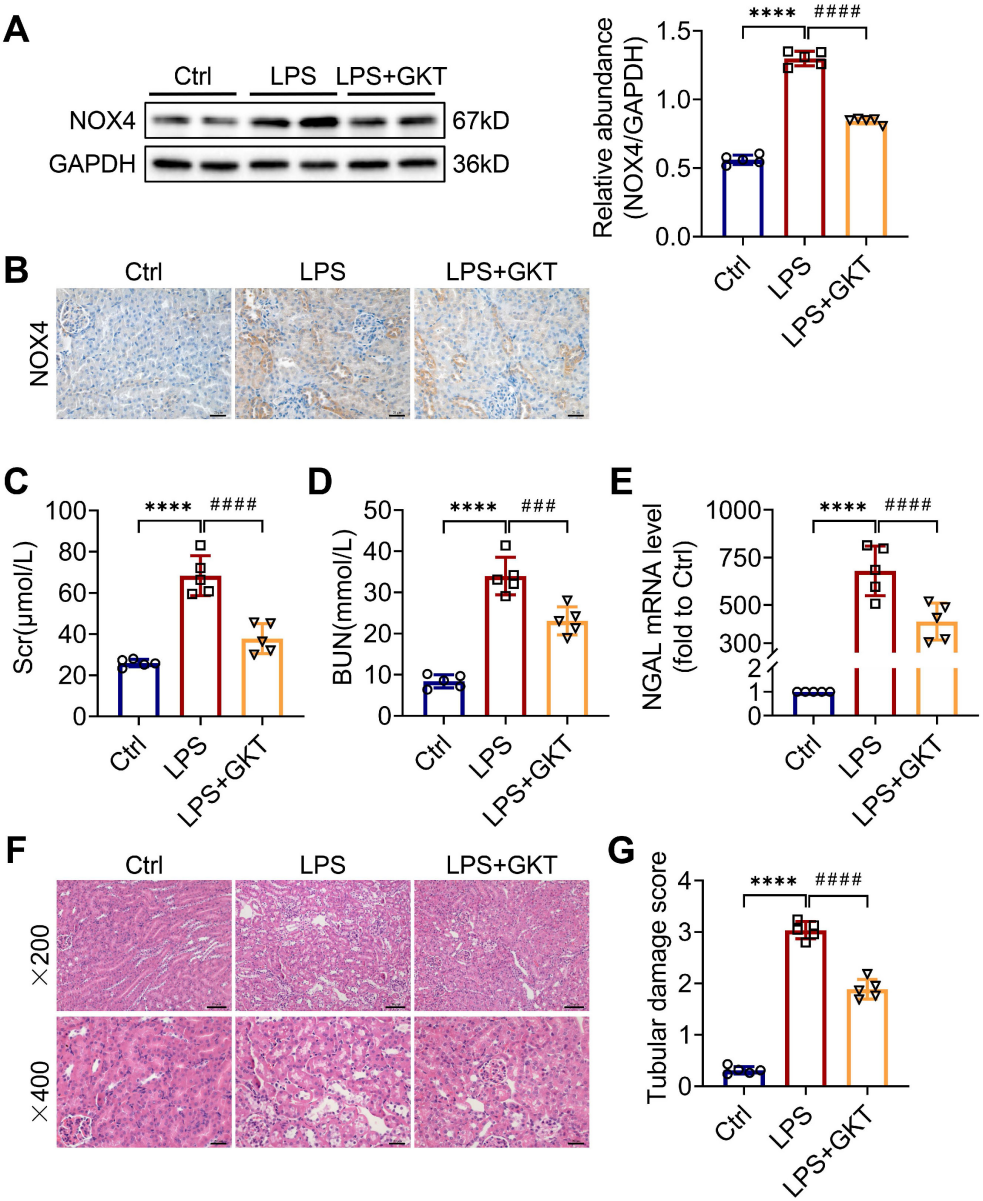
** NOX4 inhibitor GKT137831 treatment ameliorated LPS-induced S-AKI in mice.** (A) Protein expression of kidney NOX4 detected by western blot analysis and quantified by densitometry. (B) Representative micrographs of immunochemistry staining of NOX4 in kidney tissue sections (×400, scale bars = 20 μm). (C) Scr and (D) BUN levels in different groups of mice. (E) Renal NGAL mRNA expression measured by RT‒qPCR. (F) Representative images of HE staining (×200, scale bars = 50 μm; ×400, scale bars = 20 μm). (G) Tubular damage scores of kidney tissues based on HE staining. Data are represented as the mean ± SD, n = 5. ^****^P < 0.0001 vs Ctrl; ^###^P < 0.001, ^####^P < 0.0001 vs LPS.

To further investigate whether the NOX4 inhibitor GKT137831 (GKT) offers a renoprotective effect on S-AKI, the mice were administered GKT137831 orally at 60 mg/kg/d for six consecutive days prior to injection of LPS. As shown in Figure 2A-B, GKT137831 administration reduced effectively the upregulation of NOX4 expression in RTECs induced by LPS. GKT137831 treatment significantly reduced the elevation of Scr and BUN induced by LPS (Figure 2C-D) and reduced the mRNA level of NGAL in injured kidneys (Figure 2E). In addition, an obvious improvement in renal morphologic damage was observed in the GKT137831 group via HE staining (Figure 2F-G). Similarly, NOX4 expression was also inhibited by GKT137831 treatment in CLP-injured kidneys (Figure S2A). Meanwhile, the CLP-induced kidney dysfunction (Figure S2B-C) and tubular injury (Figure S2D-F) were also alleviated by GKT137831 treatment. Taken together, all these data indicated that NOX4 in RTECs was induced by sepsis, and both genetic and pharmacological suppression of NOX4 could attenuate S-AKI.

### Inhibition of NOX4 reduces mitochondrial dysfunction, inflammation and cell apoptosis in S-AKI mice

**Figure 3:**
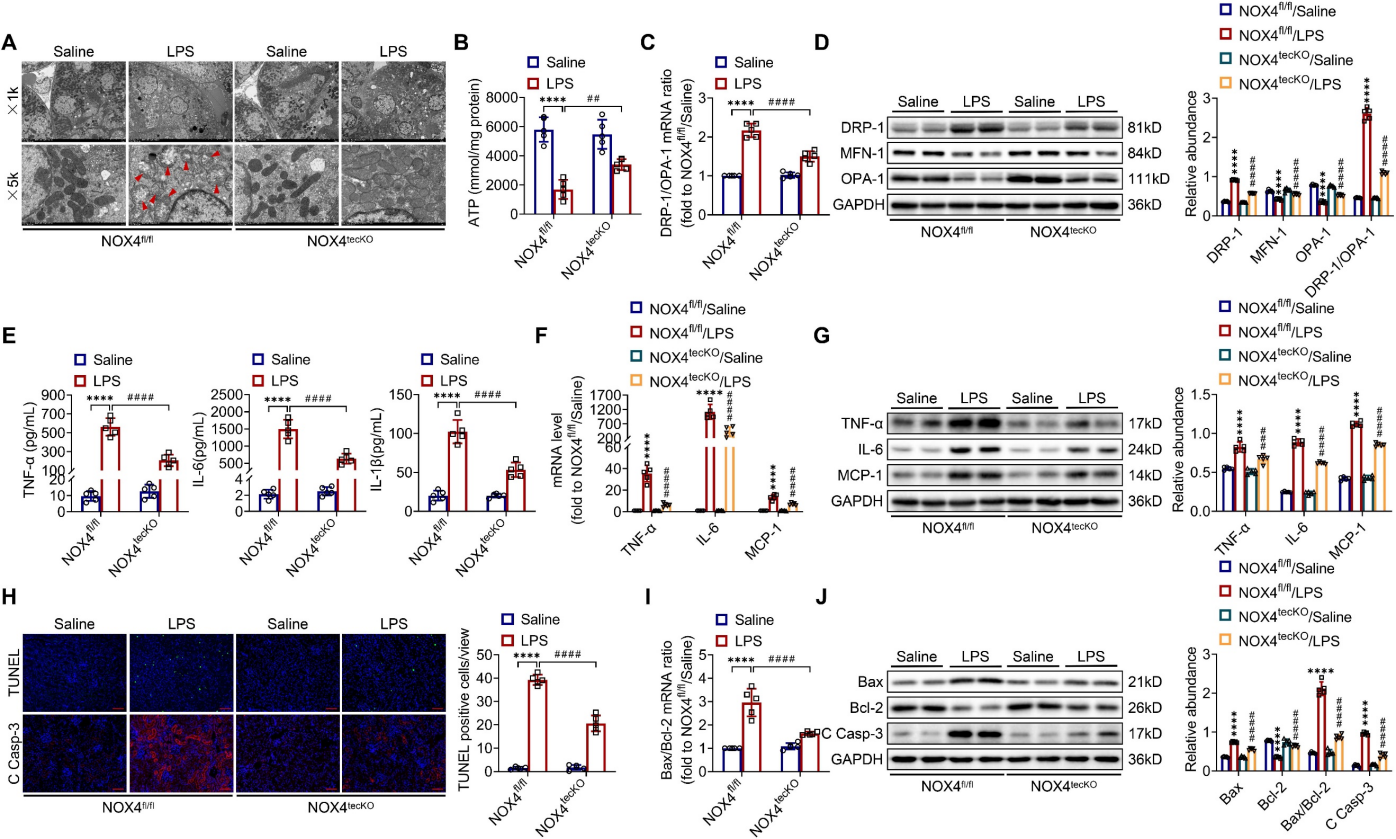
** RTEC-specific knockout of NOX4 attenuated LPS-induced kidney mitochondrial dysfunction, inflammation and cell apoptosis in mice.** (A) Representative photomicrographs of mitochondria in RTECs collected by transmission electron microscopy (×1000, scale bars = 10 μm; ×5000, scale bars = 2 μm). The red triangle indicates injured mitochondria. (B) ATP production in renal tissues detected using assay kits. (C) Gene expression ratio of the dynamic regulatory related molecules DRP-1/OPA-1 in renal tissues measured by RT‒qPCR. (D) The protein expression of dynamic regulatory-related molecules, including DRP-1, MFN-1 and OPA-1, in the kidneys was analyzed by western blot analysis and quantified by densitometry. (E) Serum levels of proinflammatory cytokines, including TNF-α, IL-6 and IL-1β, determined using ELISA kits. (F) Renal mRNA levels of proinflammatory cytokines, including TNF-α, IL-6 and MCP-1, measured by RT‒qPCR. (G) The protein expression of proinflammatory cytokines, including TNF-α, IL-6 and MCP-1, in kidneys was analyzed by western blot analysis and quantified by densitometry. (H) Representative images of TUNEL staining and immunofluorescence staining of C Casp-3 (×200, scale bars = 50 μm) and quantification of TUNEL-positive cells in the kidney cortex. (I) Gene expression ratio of apoptotic markers Bax/Bcl-2 in renal tissues measured by RT‒qPCR. (J) The protein expression of apoptotic markers, including Bax, Bcl-2 and C Casp-3, in the kidneys was analyzed by western blot analysis and quantified by densitometry. Data are represented as the mean ± SD, n = 5. ^****^P < 0.0001 vs NOX4^fl/fl^/Saline; ^##^P < 0.01, ^###^P < 0.001, ^####^P < 0.0001 vs NOX4^fl/fl^/LPS.

**Figure 4:**
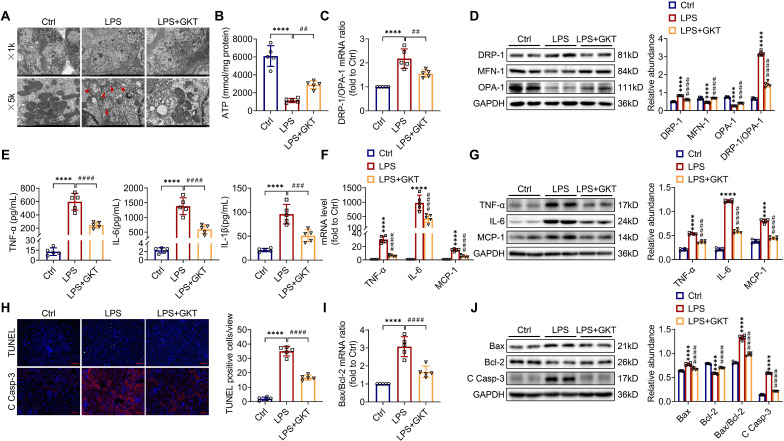
** NOX4 inhibitor GKT137831 treatment suppressed LPS-induced kidney mitochondrial dysfunction, inflammation and cell apoptosis in mice.** (A) Representative photomicrographs of mitochondria in RTECs collected by transmission electron microscopy (×1000, scale bars = 10 μm; ×5000, scale bars = 2 μm). The red triangle indicates injured mitochondria. (B) ATP production in renal tissues detected using assay kits. (C) Gene expression ratio of DRP-1/OPA-1 in renal tissues measured by RT‒qPCR. (D) DRP-1, MFN-1 and OPA-1 protein expression in the kidneys was analyzed by western blot analysis and quantified by densitometry. (E) Serum levels of TNF-α, IL-6 and IL-1β determined using ELISA kits. (F) Renal mRNA levels of TNF-α, IL-6 and MCP-1 measured by RT‒qPCR. (G) The protein expression levels of TNF-α, IL-6 and MCP-1 in kidneys were analyzed by western blot analysis and quantified by densitometry. (H) Representative images of TUNEL staining and immunofluorescence staining of C Casp-3 (×200, scale bars = 50 μm) and quantification of TUNEL-positive cells in the kidney cortex. (I) Gene expression ratio of Bax/Bcl-2 in renal tissues measured by RT‒qPCR. (J) The protein expression levels of Bax, Bcl-2 and C Casp-3 in the kidneys were analyzed by western blot analysis and quantified by densitometry. Data are represented as the mean ± SD, n = 5. ^****^P < 0.0001 vs Ctrl; ^##^P < 0.01, ^###^P < 0.001, ^####^P < 0.0001 vs LPS.

There is increasingly evidence that mitochondrial dysfunction contributes significantly to the AKI pathogenesis 22, 23. We concentrated on the morphology, function as well as dynamics balance of mitochondria. As delineated in Figure 3A and Figure 4A, we observed ultrastructural changes in RTECs of control mice after LPS, consisting of mitochondrial swelling, cristae loss, fragmentation, together with vacuoles in the mitochondrial matrix, which were obviously alleviated by RTEC-specific deletion of NOX4 or GKT137831 administration. Additionally, we found that renal ATP production of LPS mice was dramatically reduced in LPS mice compared with control mice, while NOX4 suppression restored the mitochondria ATP production capacity (Figure 3B and Figure 4B). Furthermore, the expression levels of the mitochondrial fission protein DRP-1 and the mitochondrial fusion proteins MFN-1 and OPA-1 were examined via exploiting RT-qPCR and western blotting. The findings indicated that the level of protein of DRP-1 along with the mRNA and protein ratios of DRP-1/OPA-1 were raised, while the protein levels of MFN-1 and OPA-1 were down-regulated after being treated by LPS, while genetic or pharmacological inhibition of NOX4 reversed the abovementioned abnormalities (Figure 3C-D and Figure 4C-D).

Inflammation at the site of renal tissue damage is a feature of kidney injury during sepsis 4, 5. We found that the TNF-α, IL-6 and IL-1β serum levels were evidently reduced with NOX4 deficiency or GKT137831 treatment compared with LPS mice (Figure 3E and Figure 4E). The mRNA and protein expression of TNF-α, IL-6 along with MCP-1 in injured kidneys was also downregulated by RTEC-specific knockout of NOX4 or GKT137831 treatment (Figure 3F-G and Figure 4F-G).

Moreover, cell apoptosis in renal is another prominent feature in S-AKI pathogenesis 24, 25. As shown in Figure 3H and Figure 4H, apoptotic cell nuclei were evidently noted in kidney sections from LPS mice, while genetic and pharmacological inhibition of NOX4 both suppressed renal cell apoptosis. Correspondingly, the expression levels of the proapoptotic protein Bax and the apoptosis-executing protein cleaved caspase-3 (C Casp-3) in the kidneys were downregulated by NOX4 deficiency or GKT137831 treatment, while the expression of the antiapoptotic protein Bcl-2 was enhanced (Figure 3H, J and Figure 4H, J), in addition to the decrease in Bax/Bcl-2 mRNA and protein ratios (Figure 3I-J and Figure 4I-J).

Likewise, in CLP-induced mice, we also found that NOX4 inhibition could attenuate mitochondrial malfunction (Figure S3A-B), inflammation (Figure S3C), and degree of apoptosis (Figure S3D-E) of injured kidney. In general, all these findings demonstrated that RTEC-specific deletion of NOX4 or GKT137831 treatment attenuated RTEC mitochondrial dysfunction and inflammation, as well as cell apoptosis in S-AKI.

### Inhibition of NOX4 attenuates kidney damage in S-AKI mice through suppression of ROS generation and activation of the NF-κB p65 signaling

**Figure 5:**
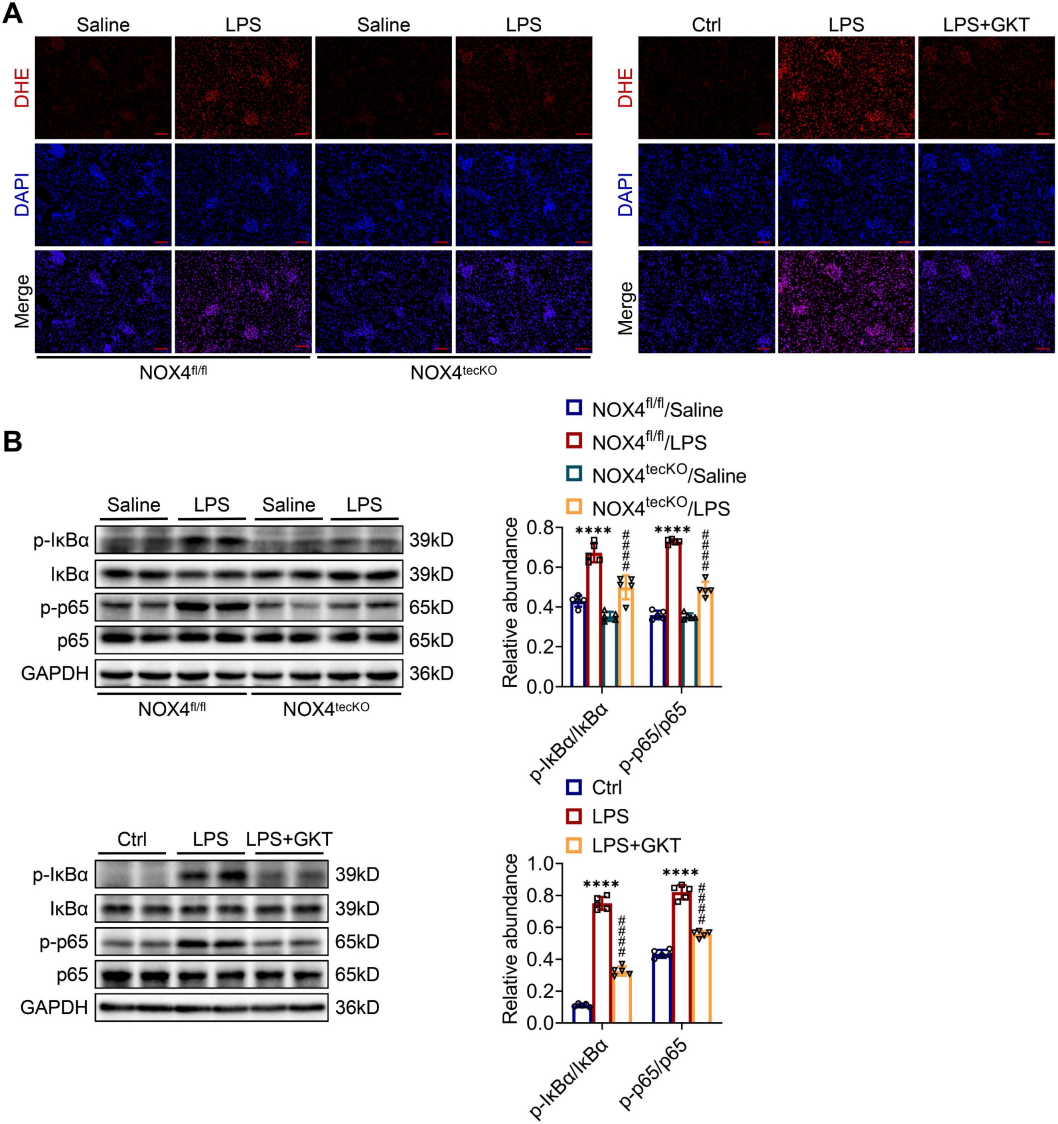
** Genetic or pharmacological inhibition of NOX4 attenuated ROS production and NF-κB p65 signaling activation in injured kidneys.** (A) ROS were assessed *in situ* by DHE staining (×200, scale bars = 50 μm). (B) The protein expression levels of p-IκBα, IκBα, p-p65 and p65 in the kidneys were analyzed by western blot analysis and quantified by densitometry. Data are represented as the mean ± SD, n = 5. ^****^P < 0.0001 for NOX4^fl/fl^/LPS vs NOX4^fl/fl^/Saline or for LPS vs Ctrl; ^####^P < 0.0001 for NOX4^tecKO^/LPS vs NOX4^fl/fl^/LPS or for LPS + GKT vs LPS.

To further examine the molecular mechanism through which NOX4 in RTECs affects S-AKI, the ROS concentrations in renal tissue were examined. The intensity of red fluorescence, indicating the levels of ROS, was markedly decreased in damaged kidneys of NOX4^tecKO^ mice in comparison to NOX4^fl/fl^ mice. GKT137831 treatment also effectively reduced the increases in ROS in the kidneys of S-AKI mice induced by LPS (Figure 5A). In addition, the phosphorylation levels for the proteins of IκBα (p-IκBα) together with NF-κB p65 (p-p65) in the kidneys were significantly downregulated by genetic knockout or pharmacological blockade of NOX4 (Figure 5B), which indicated that NOX4 suppression could inhibit the NF-κB p65 signal pathway activation. Together, the results displayed that both RTEC-specific deletion of NOX4 and GKT137831 treatment might protect LPS-induced S-AKI mice by suppressing the ROS generation and NF-κB p65 signaling pathway activation.

### Silencing NOX4 gene expression mitigates mitochondrial dysfunction, inflammation and apoptosis and in LPS-stimulated TCMK-1 cells

**Figure 6:**
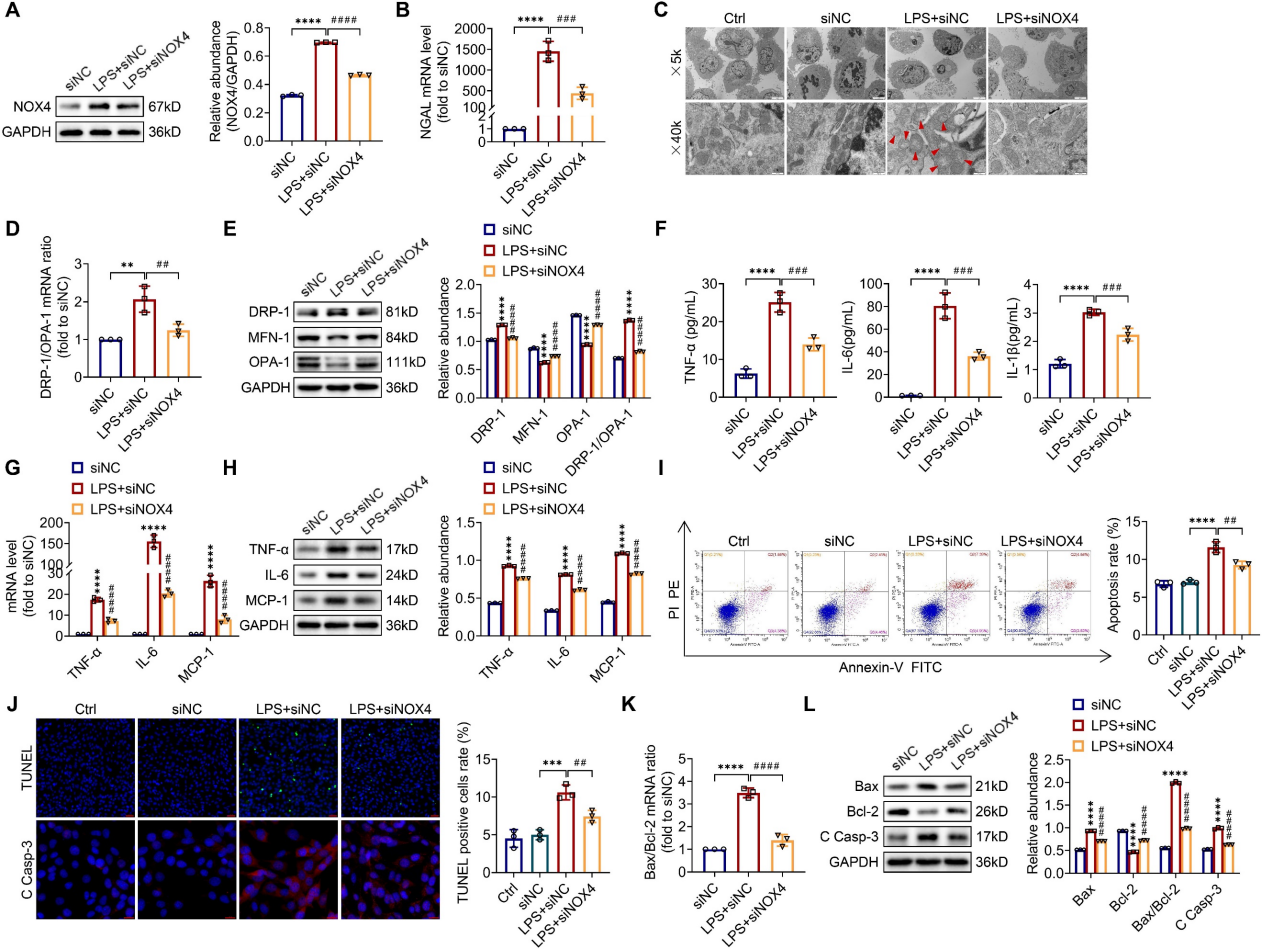
** Knocking down NOX4 inhibited mitochondrial dysfunction, inflammation and apoptosis in LPS-stimulated TCMK-1 cells.** (A) Western blot analysis and quantification by densitometry of NOX4 in each group of TCMK-1 cells. (B) NGAL mRNA expression in TCMK-1 cells measured by RT‒qPCR. (C) Representative photomicrographs of mitochondria in TCMK-1 cells collected by transmission electron microscopy (×5000, scale bars = 5 μm; ×40000, scale bars = 500 nm). The red triangle indicates injured mitochondria. (D) Gene expression ratio of DRP-1/OPA-1 in TCMK-1 cells measured by RT‒qPCR. (E) The protein expression levels of DRP-1, MFN-1 and OPA-1 in TCMK-1 cells were analyzed by western blot analysis and quantified by densitometry. (F) The cell supernatant levels of TNF-α, IL-6 and IL-1β determined using assay kits. (G) The mRNA levels of TNF-α, IL-6 and MCP-1 in TCMK-1 cells measured by RT‒qPCR. (H) The protein expression levels of TNF-α, IL-6 and MCP-1 in TCMK-1 cells were analyzed by western blot analysis and quantified by densitometry. (I) Representative flow cytometric plots of TCMK-1-cell apoptosis and quantification of the apoptosis rate. (J) Representative images of TUNEL staining (×200, scale bars = 50 μm) and immunofluorescence staining of C Casp-3 (×400, scale bars = 20 μm) and the percentage of TUNEL-positive TCMK-1 cells. (K) Gene expression ratio of Bax/Bcl-2 in TCMK-1 cells measured by RT‒qPCR. (L) The protein expression levels of Bax, Bcl-2 and C Casp-3 in TCMK-1 cells were analyzed by western blot analysis and quantified by densitometry. Data are represented as the mean ± SD, n = 3. ^**^P < 0.01, ^***^P < 0.001, ^****^P < 0.0001 vs siNC; ^##^P < 0.01, ^###^P < 0.001, ^####^P < 0.0001 vs LPS + siNC.

To explore the optimum modeling dose and timing of LPS *in vitro*, TCMK-1 cells were stimulated with LPS at doses of 0-300 μg/mL and times of 0-24 h. As presented in Figure S4, LPS induced the TLR4, NOX4 and C Casp-3 expression both in a dose- and time-related way (0-300 μg/ml and 0-24 h). In the end, we selected 100 μg/ml LPS to stimulate TCMK-1 cells for 12 h. After that, to examine whether inhibition of NOX4 with siRNA could repress mitochondrial dysfunction, inflammation, as well as apoptosis *in vitro*, TCMK-1 cells were transfected with NOX4 siRNA, which effectively knocked down NOX4 expression (Figure S5A). After LPS stimulation, the protein expression of NOX4 was upregulated significantly, while NOX4 siRNA reversed this trend (Figure 6A).

Furthermore, silencing the NOX4 gene obviously attenuated LPS-stimulated cellular injury with reduced NGAL mRNA levels in TCMK-1 cells (Figure 6B). Corresponding to the experiments* in vivo*, we observed mitochondrial morphology with a transmission electron microscope and assessed the mitochondrial dynamics balance by detecting the DRP-1, MFN-1 and OPA-1 expressions in TCMK-1 cells. The results indicated that NOX4 knockdown alleviated mitochondrial morphological destruction and reversed the changes in the protein levels of DRP-1, MFN-1 and OPA-1, along with the mRNA and protein ratios of DRP-1/OPA-1 (Figure 6C-E). The levels of TNF-α, IL-6 together with IL-1β in the supernatant were considerably reduced after NOX4 gene silencing in contrast to the LPS + siNC group. Similarly, the upregulation of TNF-α, IL-6 and MCP-1 mRNA and protein levels in TCMK-1 cells were reversed by siNOX4 transfection, which was corroborated by the *in vivo* findings (Figure 6F-H). We used flow cytometry and TUNEL staining to investigate the extent of apoptosis in TCMK-1 cells stimulated by LPS, and we showed that knocking down NOX4 reduced the apoptosis rate and TUNEL-positive cells in the LPS + siNC group (Figure 6I-J). Besides, siNOX4 administration decreased the mRNA and protein ratios of Bax/Bcl-2 (Figure 6K), decreased proteins of Bax and C Casp-3 and raised Bcl‐2 protein in comparison to the LPS + siNC group (Figure 6J, L). In total, these data proved that NOX4 was induced by LPS in TCMK-1 cells, and siNOX4 transfection attenuated mitochondrial dysfunction, inflammation and apoptosis in TCMK-1 cells with LPS simulation.

### Overexpression of NOX4 aggravates mitochondrial dysfunction, inflammation and apoptosis and in LPS-stimulated TCMK-1 cells

**Figure 7:**
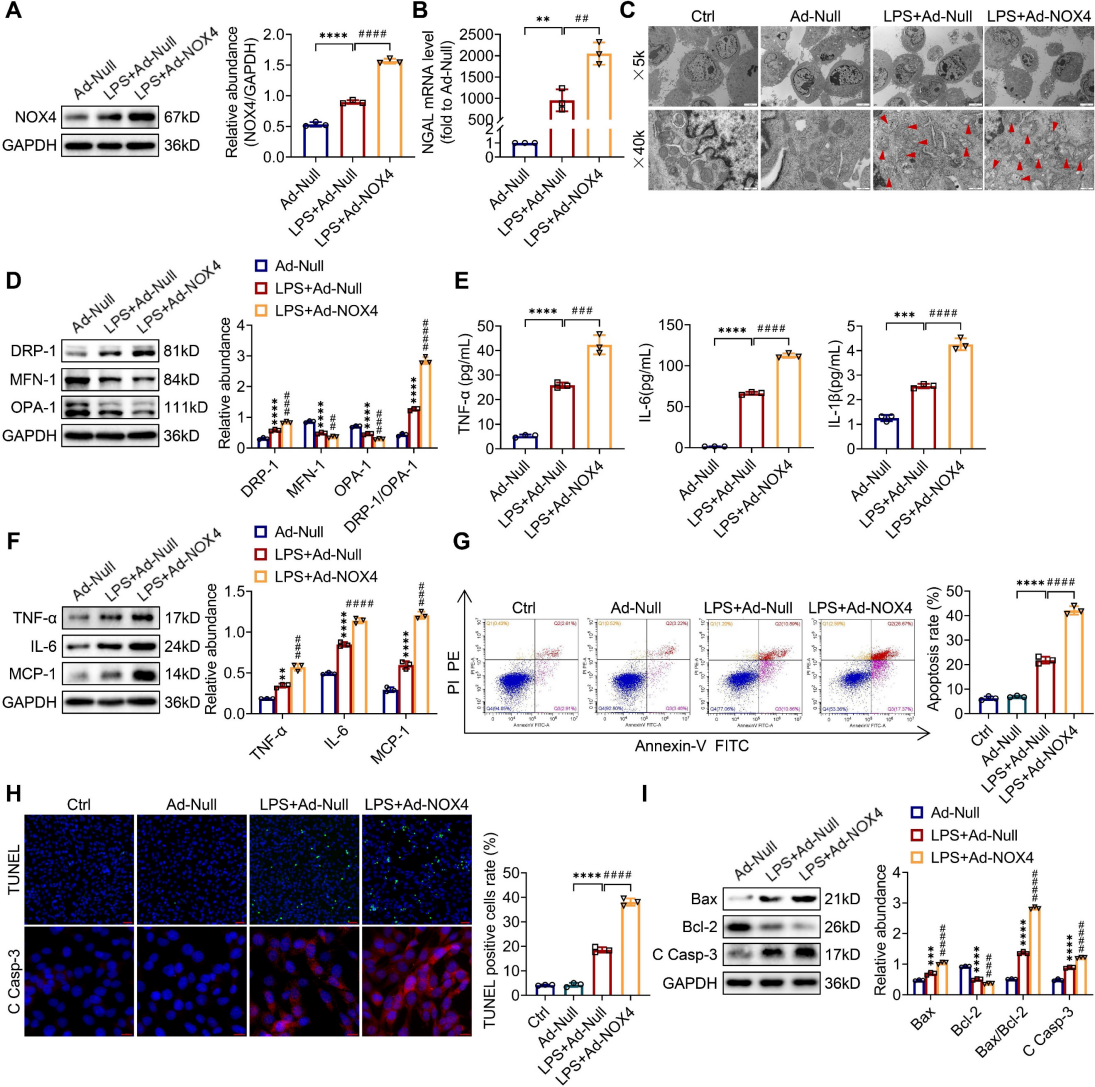
** NOX4 overexpression worsened mitochondrial dysfunction, inflammation and apoptosis in LPS-stimulated TCMK-1 cells.** (A) Western blot analysis and quantification by densitometry of NOX4 in each group of TCMK-1 cells. (B) NGAL mRNA expression in TCMK-1 cells measured by RT‒qPCR. (C) Representative photomicrographs of mitochondria in TCMK-1 cells collected by transmission electron microscopy (×5000, scale bars = 5 μm; ×40000, scale bars = 500 nm). The red triangle indicates injured mitochondria. (D) The protein expression levels of DRP-1, MFN-1 and OPA-1 in TCMK-1 cells were analyzed by western blot analysis and quantified by densitometry. (E) The cell supernatant levels of TNF-α, IL-6 and IL-1β determined using assay kits. (F) The protein expression levels of TNF-α, IL-6 and MCP-1 in TCMK-1 cells were analyzed by western blot analysis and quantified by densitometry. (G) Representative flow cytometric plots of TCMK-1-cell apoptosis and quantification of the apoptosis rate. (H) Representative images of TUNEL staining (×200, scale bars = 50 μm) and immunofluorescence staining of C Casp-3 (×400, scale bars = 20 μm) and the percentage of TUNEL-positive TCMK-1 cells. (I) The protein expression levels of Bax, Bcl-2 and C Casp-3 in TCMK-1 cells were analyzed by western blot analysis and quantified by densitometry. Data are represented as the mean ± SD, n = 3. ^**^P < 0.01, ^***^P < 0.001, ^****^P < 0.0001 vs Ad-Null; ^##^P < 0.01, ^###^P < 0.001, ^####^P < 0.0001 vs LPS + Ad-Null.

To examine whether the overexpression of NOX4 by adenoviruses could worsen mitochondrial dysfunction, inflammation along with apoptosis *in vitro*, TCMK-1 cells were transfected with Ad-NOX4, which effectively overexpressed NOX4 (Figure S5B). After LPS stimulation, the of NOX4 protein expression was raised and further increased by Ad-NOX4 transfection (Figure 7A). Overexpressing the NOX4 gene further injured LPS-stimulated TCMK-1 cell by increasing NGAL mRNA levels (Figure 7B). In terms of mitochondrial homeostasis, mitochondrial morphology was further destroyed (Figure 7C), and excessive mitochondrial fission was further exacerbated (Figure 7D) by Ad-NOX4 transfection. Moreover, NOX4 overexpression obviously aggravated inflammation and apoptosis stimulated by LPS, as confirmed with further increases in proinflammatory cytokines (namely, TNF-α, IL-6, IL-1β and MCP-1) (Figure 7E-F), apoptotic rate by flow cytometry and TUNEL staining (Figure 7G-H), proapoptotic markers (Bax and C Casp-3) and a further decrease in antiapoptotic markers (Bcl-2) (Figure 7H-I).

### GKT137831 treatment ameliorates mitochondrial dysfunction, inflammation and apoptosis and in LPS-stimulated TCMK-1 cells

**Figure 8:**
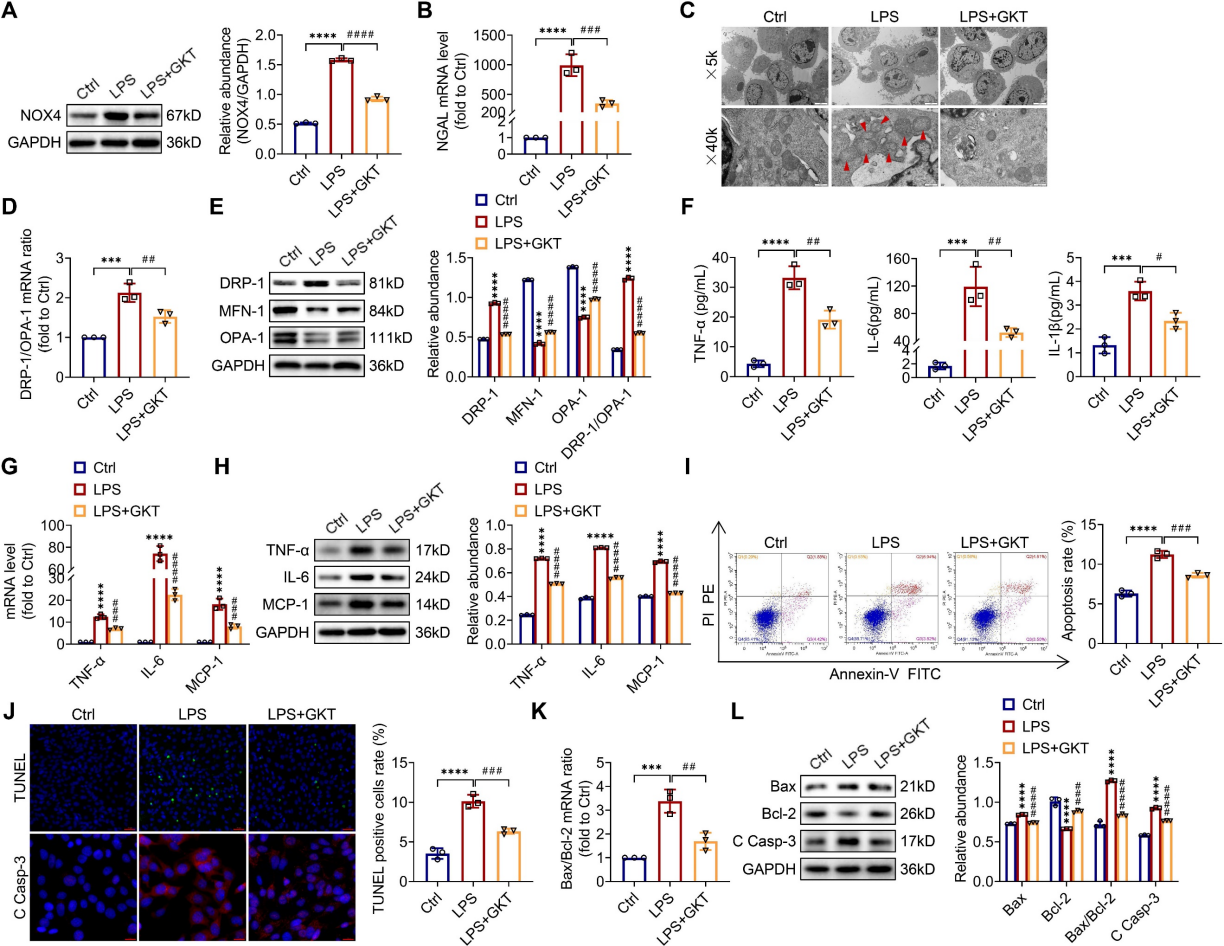
** NOX4 inhibitor GKT137831 treatment suppressed mitochondrial dysfunction, inflammation and apoptosis in LPS-stimulated TCMK-1 cells.** (A) Western blot analysis and quantification by densitometry of NOX4 in each group of TCMK-1 cells. (B) NGAL mRNA expression in TCMK-1 cells measured by RT‒qPCR. (C) Representative photomicrographs of mitochondria in TCMK-1 cells collected by transmission electron microscopy (×5000, scale bars = 5 μm; ×40000, scale bars = 500 nm). The red triangle indicates injured mitochondria. (D) Gene expression ratio of DRP-1/OPA-1 in TCMK-1 cells measured by RT‒qPCR. (E) The protein expression levels of DRP-1, MFN-1 and OPA-1 in TCMK-1 cells were analyzed by western blot analysis and quantified by densitometry. (F) The cell supernatant levels of TNF-α, IL-6 and IL-1β determined using assay kits. (G) The mRNA levels of TNF-α, IL-6 and MCP-1 in TCMK-1 cells measured by RT‒qPCR. (H) The protein expression levels of TNF-α, IL-6 and MCP-1 in TCMK-1 cells were analyzed by western blot analysis and quantified by densitometry. (I) Representative flow cytometric plots of TCMK-1-cell apoptosis and quantification of the apoptosis rate. (J) Representative images of TUNEL staining (×200, scale bars = 50 μm) and immunofluorescence staining of C Casp-3 (×400, scale bars = 20 μm) and the percentage of TUNEL-positive TCMK-1 cells. (K) Gene expression ratio of Bax/Bcl-2 in TCMK-1 cells measured by RT‒qPCR. (L) The protein expression levels of Bax, Bcl-2 and C Casp-3 in TCMK-1 cells were analyzed by western blot analysis and quantified by densitometry. Data are represented as the mean ± SD, n = 3. ^***^P < 0.001, ^****^P < 0.0001 vs Ctrl;^ #^P < 0.05, ^##^P < 0.01, ^###^P < 0.001, ^####^P < 0.0001 vs LPS.

For investigating the optimum concentration of GKT137831 *in vitro*, CCK-8 assay was employed to investigate the cytoprotective effect of GKT137831 (0-40 μM) in TCMK-1 cells and observed that 10 μM GKT137831 provided the most significant improvement in cell viability in in the TCMK-1 cells simulated with LPS (Figure S6). As a result, 10 μM GKT137831 was applied for the subsequent experiments. GKT137831 showed significant suppression of NOX4 expression in the TCMK-1 cells induced with LPS (Figure 8A). GKT137831 also exhibited a protective effect against cellular injury (Figure 8B), mitochondrial dysfunction (Figure 8C-E) and inflammation (Figure 8F-H), as well as apoptosis (Figure 8I-L) in the TCMK-1 cells simulated by LPS.

### ROS production and NF-κB p65 pathway activation were modulated by NOX4 expression in TCMK-1 cells stimulated by LPS

**Figure 9:**
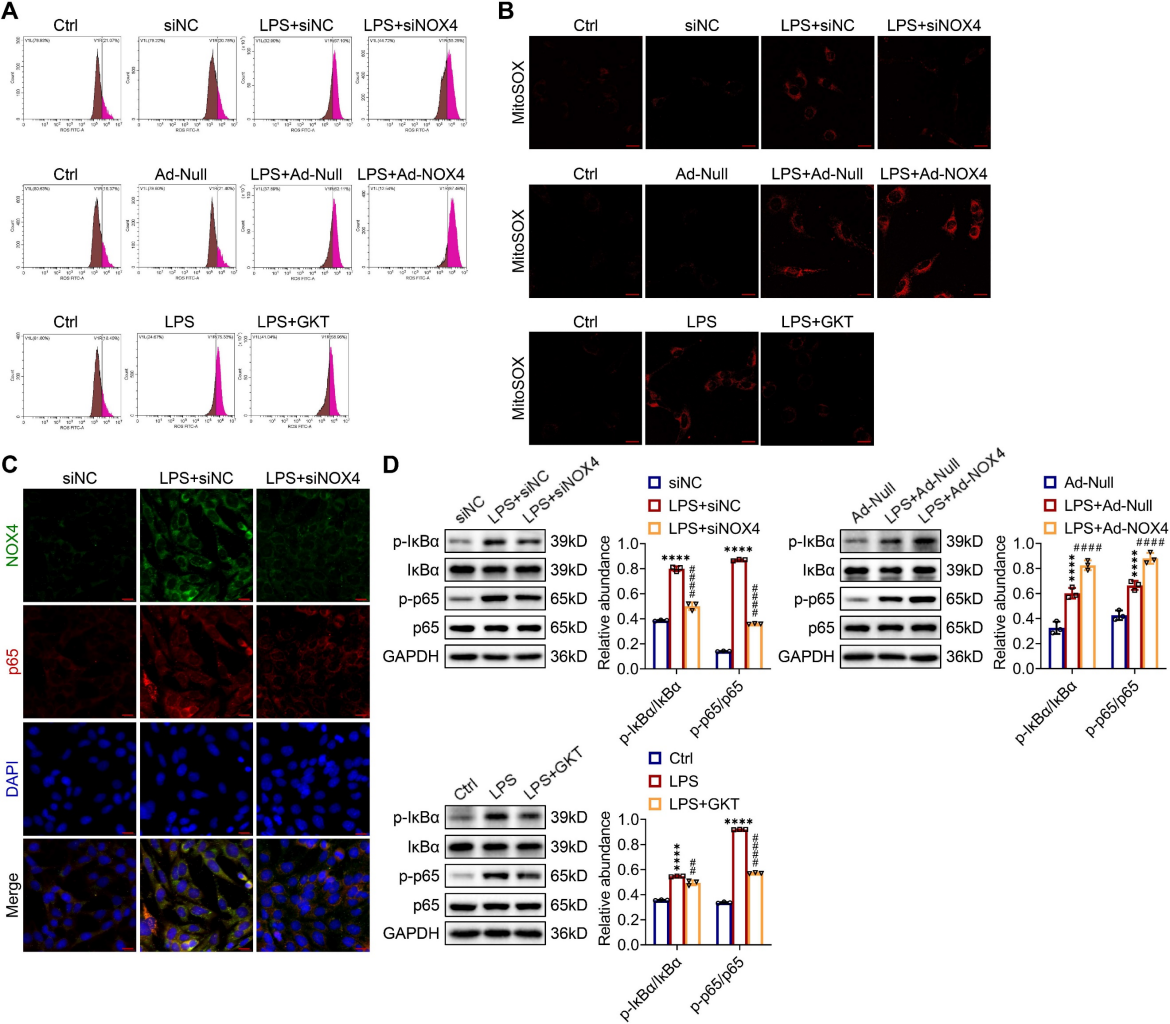
** NOX4 expression modulated ROS production and NF-κB p65 signaling activation in LPS-stimulated TCMK-1 cells.** (A) ROS levels in TCMK-1 cells were determined by flow cytometry. (B) Representative confocal images of MitoSOX staining in TCMK-1 cells (×630, scale bars = 20 μm). (C) Representative micrographs of double immunofluorescence staining of NOX4 (green) and p65 (red) in TCMK-1 cells (×400, scale bars = 20 μm). (D) The protein expression levels of p-IκBα, IκBα, p-p65 and p65 in TCMK-1 cells were analyzed by western blot analysis and quantified by densitometry. Data are represented as the mean ± SD, n = 3. ^****^P < 0.0001 for LPS + siNC vs siNC, LPS + Ad-Null vs Ad-Null, or LPS vs Ctrl; ^##^P < 0.01, ^####^P < 0.0001 for LPS + siNOX4 vs LPS + siNC, LPS + Ad-NOX4 vs LPS + Ad-Null, or LPS + GKT vs LPS.

As shown in Figure 9A, the ROS concentrations in LPS-stimulated TCMK-1 cells were significantly elevated. Genetic and pharmacological inhibition of NOX4 both effectively diminished the increases in ROS, while overexpression of NOX4 further enhanced the production of ROS *in vitro*. In terms of mtROS production, the MitoSOX staining outcomes showed consistent trends with ROS levels in TCMK-1 cells (Figure 9B).

As indicated by double immunofluorescence staining, the expression and nuclear transport of NF-κB p65 were obviously inhibited by NOX4 silencing in TCMK-1 cells with LPS simulation (Figure 9C). The p-IκBα and p-p65 proteins were effectively suppressed by NOX4 knockdown or GKT137831 administration in in TCMK-1 cells simulated by LPS, while NOX4 overexpression further upregulated the p-IκBα and p-p65 protein levels (Figure 9D). In summary, these findings indicated that the NOX4 expression could influence ROS generation and activation of NF-κB p65 signaling.

## Discussion

In the current study, we explored the role of NOX4 in the pathophysiology of septic AKI *in vivo* and *in vitro*. Genetic knockout/silencing or pharmacological blockade of NOX4 protected mouse kidney and TCMK-1 cells through the attenuation of oxidative stress, mitochondrial morphology/function/dynamics balance and inflammation. Conversely, overexpression of NOX4 with adenoviruses worsened TCMK-1-cell oxidative injury, mitochondrial dysfunction and inflammatory injury under LPS/CLP challenge. NOX4 inhibition might be a prospective therapeutic strategy for the future S-AKI treatment.

In comparison to the other categories of AKI, septic AKI exhibits just modest histological changes in spite of a substantial decrease in function 26. As the mechanism of S-AKI remains unclear, current treatments are mainly supportive and are not targeted toward its pathophysiology. S-AKI has been traditionally considered secondary to ischemic injury during septic shock, but experimental observations have shown that S-AKI can occur in the setting of renal hyperemia, and that ischemia is not necessary for the decrease of glomerular filtration rate (GFR) to occur 27. S-AKI is both considered to be a failure of energy status and also as a combined response involving an inflammatory cascade, transcriptional events, metabolic disturbance and apoptosis 28. During systemic inflammatory response syndrome (SIRS), there is increased production of ROS, which subsequently results in microcirculatory abnormalities, localized tissue hypoxia and mitochondrial dysfunction, triggering a vicious cycle of cellular infection causing AKI 29.

NOX4 is a kind of ROS synthesis protein expressed in renal tubular epithelial cells 30, renal interstitial fibroblasts 13 and infiltrated cells 31, which exerts a significant role in modulating the physiological activities of protein, lipid, DNA and transcription in the kidney 32. Previous researches have confirmed that NOX4-mediated ROS plays a critical role during the process of renal fibrosis in chronic kidney diseases such as diabetic nephropathy 33. In recent years, growing evidence has also suggested that NOX4 is also implicated in AKI pathogenesis 34, 35. Our past studies found that NOX4 was inherently expressed in the mouse renal tubular epithelial cells, while the increased NOX4 was downregulated after anti-inflammatory treatment with Maresin 1 by suppressing the activation of the NOX4/ROS/NF-κB signals 20. As a major contributor of ROS in the kidney, NOX4 is a promising potential therapeutic candidate for protection against oxidative stress injury in kidney disease.

The changes in NOX4 and its upstream/downstream signaling pathway have been studied in different AKI models. In the IR-AKI model, hypoxia induces apoptosis in HK-2 cells by inducing NOX4-dependent ROS production via TGF-β-Smad pathway, and pretreatment with the NOX4 inhibitor GKT137831 attenuated renal structural damage and reduced apoptotic cells 14. A research further confirmed that Smad3 bound to the promoter region of NOX4 and induced ROS production and inflammation 16. Other studies showed that the renoprotective effects of different remedies, such as ellagic acid (EA) and lysine-specific demethylase 1 (LSD1), were achieved by suppressing the NOX4/JAK/STAT and TLR/NOX4 signaling pathways in IR-AKI 36, 37. In a model of cisplatin-induced AKI, NOX4 aggravated nephrotoxicity induced by cisplatin through facilitating ROS-mediated programmed inflammation and cell death 15. In a rat model of colistin-induced AKI, the expression of NOX4 in HK-2 cells was significantly increased following colistin exposure. Knockdown of NOX4 transcription reduced ROS generation, lowered the levels of pro-inflammatory markers and attenuated apoptosis 38. A similar renoprotective role of NOX4 inhibition by decreasing ROS and reducing inflammation was also observed in contrast-induced AKI 39.

The raised generation of ROS causes the tissue damage observed in LPS-exposed animals. Inhibition of NOX4 with apocynin significantly reduced the ROS level stimulated by LPS administration 40. In LPS-induced AKI, dexmedetomidine attenuated LPS-induced AKI via suppressing oxidative stress damage and nucleotide-binding domain-like receptor protein 3 (NLRP3) inflammasome activation by modulating the TLR4/NOX4/NF-κB pathway 41. Natural compounds such as hispidulin and shikonin were found to improve kidney function in S-AKI by downregulating NOX4 42 and modulating the NOX4/PTEN/AKT pathway 43. To date, few researches have concentrated on the therapeutic effects of targeting NOX4 on S-AKI. For the first time, we showed that direct inhibition of NOX4 by either genetic knockout/silencing or pharmacological antagonist could effectively ameliorate oxidative stress and inflammatory signal NF-κB, reduce mitochondrial malfunction and apoptosis, and preserve renal function both *in vivo* and *in vitro*. Mechanistically, cytosolic proteins are required for the regulation of NOX isozymes. Src homology 3 (SH3) domain-containing YSC84-like 1 (SH3YL1), a NOX4 cytosolic regulator, mediates LPS-induced H_2_O_2_ generation through the formation of a ternary complex of p22 (phox)-SH3YL1-NOX4, which ultimately leads to AKI 19. In kidney tubular epithelial cells, receptor-interacting protein kinase-3 (RIPK3) facilitated oxidative stress, and mitochondrial dysfunction implicating NOX4 and RIPK3 upregulation was necessary for elevated mitochondrial translocation of NOX4 in sepsis 44. The detailed interaction between NOX4 and signals related to inflammation, metabolism and apoptosis at the transcriptional and epigenetic modification levels warrants in-depth investigation.

ROS play an important role in diverse physiological processes, including cell defense, hormone synthesis, signal transduction, G protein-coupled receptors activation, etc. Since NOX4 is the main source of ROS in tissues and cells, it is not surprising that NOX4 could be a double-edged sword, baring conflicting results. Unlike the aforementioned studies suggesting NOX4's pathogenicity, in an earlier study on IR-AKI, NOX4 knockout mice exhibited reduced renal function along with worse tubular apoptosis and pathological damage scores than wild-type mice 45. The NOX4-regulated pathway strongly enhanced cell survival and sustained translation initiation factor 2α (eIF2α) phosphorylation to protect against IR-AKI 46. Although NOX4 inhibition may provide a remedy for AKI, a careful assessment of the underlying adverse effects of NOX4 inhibition in different clinical settings of AKI is needed 47.

The limitations of this study are as follows. First, although we found that NOX4 inhibition effectively protected against S-AKI via inhibiting ROS and NF-κB signal, its specific molecular mechanism at the genetic/transcriptional level remains to be further elucidated. Second, we administered the NOX4 inhibitor GKT137831 before LPS injection and CLP, but in the clinic, it is difficult to practice intervention before the initial insult of S-AKI. Determining the optimal timing of NOX4 pharmaceutical inhibition is in the pipeline of our work and others. Nevertheless, the present study underlines the potential of NOX4 as a novel therapeutic target for S-AKI, which is worth future research.

In summary, our research confirmed that NOX4 in RTECs mediated LPS/CLP-induced renal tubular injuries, thus serving a vital role in the development of S-AKI. Mechanistically, NOX4 may induced the activation of ROS and NF-κB p65 signaling pathway *in vivo* and *in vitro*, which enhanced the disruption of mitochondrial homeostasis and inflammatory response, together with cell apoptosis. Therefore, modulating NOX4 expression may constitute a new therapeutic strategy for S-AKI. Mechanistic studies are required to elucidate the molecular-level regulation of NOX4 on the pathophysiological processes of S-AKI, as well as the timing and intensity of intervention.

## Supplementary Material

Supplementary methods, figures and tables.

## Abbreviations

S-AKI: septic acute kidney injury
NADPH: nicotinamide adenine dinucleotide phosphate
NOX4: NADPH oxidase 4
RTECs: renal tubular epithelial cells
LPS: lipopolysaccharide
CLP: cecal ligation and puncture
TCMK-1: mouse kidney tubular epithelium
ROS: reactive oxygen species
NF-κB: nuclear factor-kappa B
NC: negative control
Ad: adenoviruses
Scr: serum creatinine
BUN: blood urea nitrogen
FBS: fetal bovine serum
DAPI: 4,6-diamidino-2-phenylindole
LTL: lectin
HE: hematoxylin-eosin
TUNEL: terminal deoxynucleotidyl transferase‐mediated dUTP nick-end labeling
CCK-8: cell counting kit 8
ATP: Adenosine triphosphate
TNF-α: tumor necrosis factor-α
IL: interleukin
RT-qPCR: quantitative real-time polymerase chain reaction
GAPDH: glyceraldehyde phosphate dehydrogenase
NGAL: neutrophil gelatinase-associated lipocalin
DRP-1: dynamin-related protein-1
MFN-1: mitofusin-1
OPA-1: optic atrophy-1
MCP-1: monocyte chemoattractant protein-1
Bcl-2: B-cell lymphoma-2
Bax: Bcl2-Associated X
C Casp-3: cleaved capase-3
IκBα: inhibitor of kappa B alpha
