# Supplementary material for: NOX4 is a potential therapeutic target in septic acute kidney injury by inhibiting mitochondrial dysfunction and inflammation
Source: Theranostics. 2023 May 8;13(9):2863–78. doi: 10.7150/thno.81240 (PMC10240817; doi:10.7150/thno.81240)
Supplement: Supplementary file 1 — Supplementary methods, figures and tables. [file thnov13p2863s1.pdf]

Supplementary Materials

NOX4 is a potential therapeutic target in septic acute kidney injury by inhibiting mitochondrial dysfunction and inflammation

Jiameng Li[1], Liya Wang[2], Bo Wang[2], Zhuyun Zhang[2], Luojia Jiang[3], Zheng Qin[2], Yuliang Zhao[2*], Baihai Su[2]

[1]Center of Gerontology and Geriatrics, West China Hospital, Sichuan University, Chengdu 610041, China.

[2]Department of Nephrology, West China Hospital, Sichuan University, Chengdu 610041, China.

[3]Department of Nephrology, Jiujiang No. 1 People's Hospital, Jiujiang 332000, China.

[*]Corresponding author: Yuliang Zhao (E-mail: zhaoyuliang@scu.edu.cn).

Supplementary Table of Contents

1. Supplementary Methods

Animals and treatments, Cell siRNA transfection and Adenoviral transfection.

2. Supplementary Figures

Figure S1. Generation of renal tubular epithelial cell-specific (RTEC-specific) NOX4 knockout mice.

Figure S2. Genetic or pharmacologic inhibition of NOX4 ameliorated CLP-induced S-AKI in mice.

Figure S3. Genetic or pharmacologic inhibition of NOX4 alleviated CLP-induced kidney mitochondrial dysfunction, inflammation and cell apoptosis in mice.

Figure S4. Effect of doses and timing of LPS stimulation in TCMK-1 cells.

Figure S5. Western blot analysis of NOX4 knockdown by siRNA and over-expression by adenovirus.

Figure S6. The cytoprotective effect of GKT137831 on LPS-stimulated TCMK-1 cells for 12 h.

3. Supplementary Tables

Table S1. Primary antibodies used in the experiments.

Table S2. Sequences of the primers for quantitative real-time PCR.

1. Supplementary Methods

Animals and treatments

6-8-week-old male C57BL/6J mice (18-25 g) were obtained from Chengdu Dossy Experimental Animals (Chengdu, China). $NOX4^{flox/flox}$ ($NOX4^{fl/fl}$) and renal tubular epithelial cell-specific (RTEC-specific) conditional NOX4 knockout ($Cdh16\text{-}Cre^{+}$ $NOX4^{fl/fl}$, $NOX4^{tecKO}$) mice in C57BL/6J background were purchased from GemPharmatech Co.,Ltd. (Nanjing, China). The construction of $NOX4^{fl/fl}$ mice is based on CRISPR/Cas9-stimulated homologous recombination. Briefly, exon 3 and exon 4 of the NOX4 gene were flanked by two LoxP elements. Two heterozygous recombinant embryonic stem cells clones screened by homologous recombination were identified and microinjected into blastocysts from C57BL/6J mice to generate floxed heterozygous mice ($NOX4^{flox/+}$). $NOX4^{flox/+}$ mice were then inbred to obtain homozygous NOX4-floxed mice ($NOX4^{fl/fl}$). To generate $NOX4^{tecKO}$ mice, $NOX4^{fl/fl}$ mice were crossed with Cdh16-Cre mice. The genotype of $NOX4^{tecKO}$ mice was confirmed by PCR assay using specific primers (Figure S1B). Littermates carried the $NOX4^{fl/fl}$ transgene were used as controls. Mice were housed at the Animal Experiment Center of West China Hospital, Sichuan University (Chengdu, China). The mice were adapted to the environment for one week before further research.

To establish lipopolysaccharide (LPS) model, the mice (male, aged between 6-8 weeks, with a weight of 18-25 g) were intraperitoneal injected with LPS at a dose of 10 mg/kg body weight to induce septic AKI [1], and control mice were injected with the same volume of 0.9% saline. To establish cecal ligation and puncture (CLP) model, mice were anesthetized by intraperitoneal injection of pentobarbital (50 mg/kg). After disinfecting the abdomen, a middle abdominal incision (1-2 cm) was performed to expose the cecum. The cecum was ligated at 1 cm away from the blind end with a 4-0

silk suture and subsequently punctured twice using a 20-gauge needle. After gently squeezing the cecum, a small amount of feces was extruded from the perforation, the cecum was then returned to the abdominal cavity and the abdomen was closed using sterile suture in the end. Sham mice performed the same operation without ligation and puncture. All mice were resuscitated subcutaneously with 1 ml prewarmed saline after surgery. NOX4 inhibitor GKT137831 (GKT) was dissolved in 2% DMSO, 2% Tween 80, 30% PEG300 and ddH_2O in sequence. Mice in LPS + GKT group were intragastric administrated with GKT137831 solution at a dose of 60 mg/(kg·d) for 7 consecutive days before LPS injection or CLP according to the previous study and our pre-experiment [2, 3]. The mice anesthetized by pentobarbital sodium (50 mg/kg) intraperitoneal injection and sacrificed humanely at 12 h after LPS injection, then blood samples and kidney tissues were harvested and stored at -80 °C for further experiments.

Cell siRNA transfection

Transient transfections of the TCMK-1 cells with siRNAs were conducted with transfection reagent Lipofectamine^{TM} 2000 Reagent (Invitrogen, CA, USA) according to the manufacturer's instruction. The sequences of NOX4 siRNA were as follows: sense 5'-CCAUUUGCAUCGAUACUAA-3' and antisense 5'-UUAGUAUCGAUGCAAAUGG-3' (RiboBio, Guangzhou, China).

TCMK-1 cells were seeded into 6-well plates and maintained in MEM/EBSS medium (Gibco, NY, USA) supplemented with 10% fetal bovine serum (FBS) (Hyclone, Beijing, China) for 24 h. Then TCMK-1 cells were transfected with a final concentration of 100 nM siRNA using Lipofectamine^{TM} 2000 Reagent in MEM/EBSS medium without FBS. 6-8 h after transfection, the cells were subjected to fresh

medium with 10% FBS. 24 h after transfection, TCMK-1 cells were treated with 100 μg/mL LPS for 12 h.

Adenoviral transfection

TCMK-1 cells were transfected with adenoviruses (Ad) harboring NOX4 (Ad-NOX4) or no NOX4 (Ad-Null) (Hanbio Tech, Shanghai, China). The recombinant adenoviruses were prepared using the AdEasy system. Ad-Null has no transgene and was used as a control for Ad-NOX4. The titers of adenoviruses employed in this study were 1×10^{10} PFU/mL.

TCMK-1 cells were seeded into 6-well plates and maintained in MEM/EBSS medium supplemented with 10% FBS for 24 h. Then culture media were replaced by MEM/EBSS medium without FBS, and monolayers were infected with adenoviruses at a multiplicity of infection (MOI) of 300 particles/cell. 6-8 h after adenoviral transfection, the cells were subjected to fresh medium with 10% FBS. 24 h after adenoviral transfection, TCMK-1 cells were treated with 100 μg/mL LPS for 12 h.

Supplementary References

1. Wang Y, Zhu J, Liu Z, Shu S, Fu Y, Liu Y, et al. The PINK1/PARK2/optineurin pathway of mitophagy is activated for protection in septic acute kidney injury. Redox Biol. 2021; 38: 101767.

2. Aoyama T, Paik YH, Watanabe S, Laleu B, Gaggini F, Fioraso-Cartier L, et al. Nicotinamide adenine dinucleotide phosphate oxidase in experimental liver fibrosis: GKT137831 as a novel potential therapeutic agent. Hepatology. 2012; 56: 2316-27.

3. Deliyanti D, Wilkinson-Berka JL. Inhibition of NOX1/4 with GKT137831: a potential novel treatment to attenuate neuroglial cell inflammation in the retina. J

Neuroinflammation. 2015; 12: 136.

2. Supplementary Figures

A

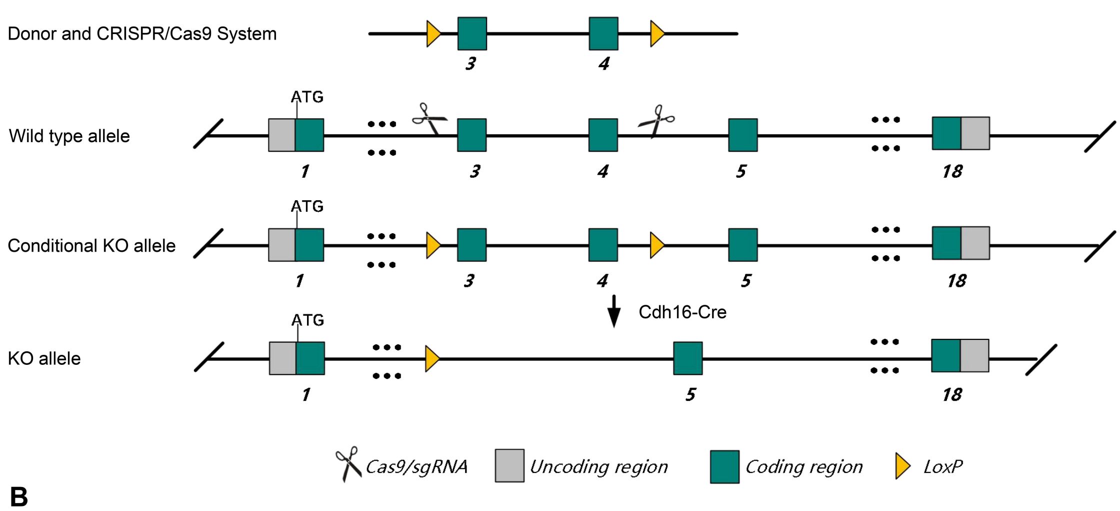

B

Primer name	Sequence (5'-3')	PCR size	Primer illustration
JS01996-Nox4-5wt-tF2	GATCAGGGAAACACTCAAGGGG	WT:262bp<br>Fl:364bp	NOX4
JS01996-Nox4-5wt-tR2	CCTAGATGGAAGATGCCTGAGATC		
H11-wt-tF1	CAGCAAAACCTGGCTGTGGATC	WT:412bp<br>KI:3421bp	Cdh16-Cre
H11-wt-tR1	ATGAGCCACCATGTGGGTGTC		

Figure S1. Generation of renal tubular epithelial cell-specific (RTEC-specific) NOX4 knockout mice. (A) Schematic of NOX4$^{flox/flox}$ (NOX4$^{fl/fl}$) mice generation by CRISPR/Cas9-stimulated homologous recombination and design strategy of RTEC-specific NOX4 knockout (NOX4^{tecKO}) mice. (B) Primer sequences used in PCR assay of the genotype of NOX4$^{fl/fl}$ mice and NOX4^{tecKO} (Cdh16-Cre^{+} NOX4$^{fl/fl}$) mice.

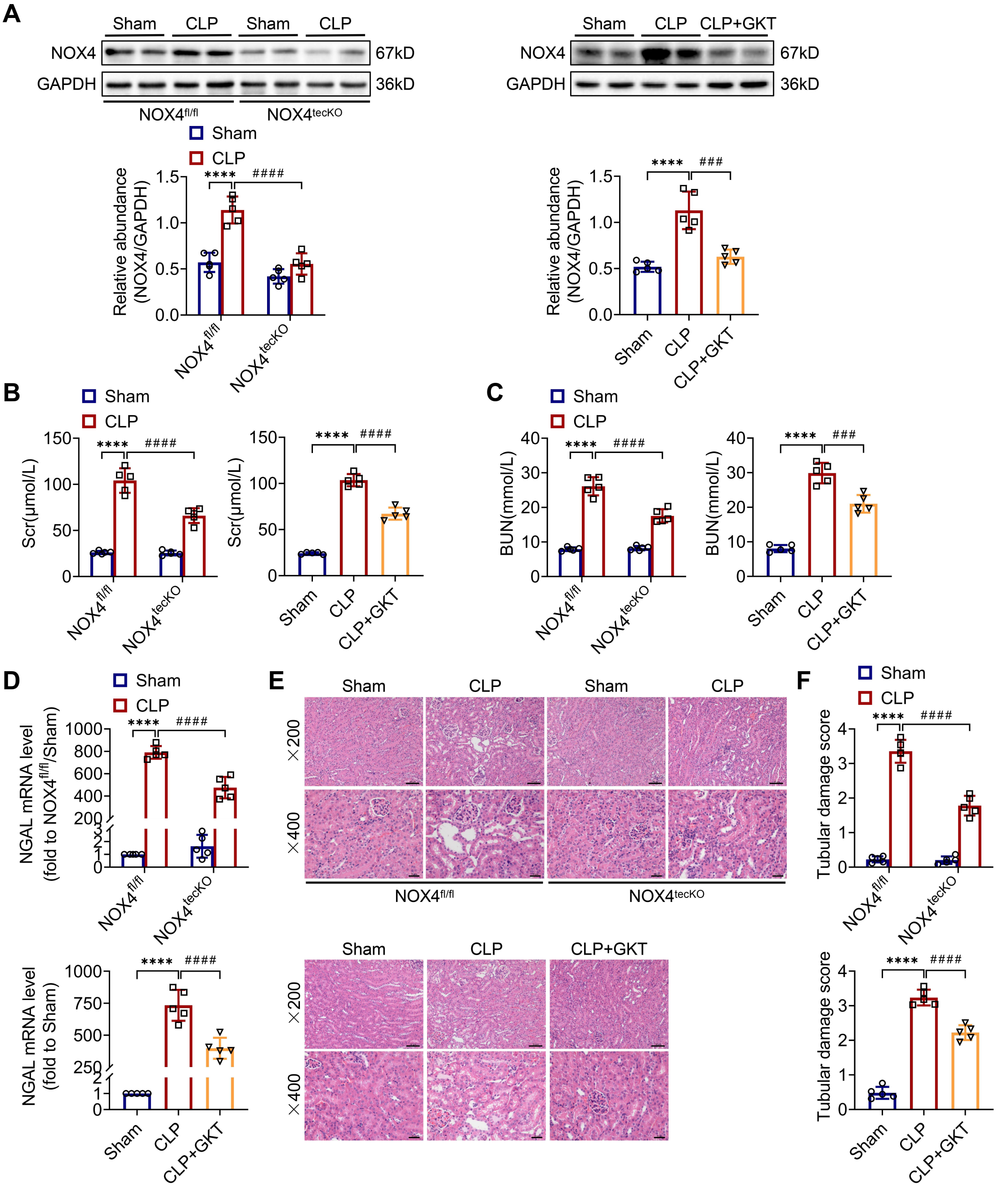

Figure S2. Genetic or pharmacologic inhibition of NOX4 ameliorated CLP-induced S-AKI in mice. (A) Protein expression of kidney NOX4 detected by western blot analysis and quantified by densitometry. (B) Scr and (C) BUN levels in different groups of mice. (D) Renal NGAL mRNA expression measured by RT–qPCR. (E) Representative images of HE staining (×200, scale bars = 50 μm; ×400, scale bars = 20 μm). (F) Tubular damage scores of kidney tissues based on HE staining.

Data are represented as the mean ± SD, n = 5. ****P < 0.0001 for NOX4$^{fl/fl}$/CLP vs NOX4$^{fl/fl}$/Sham or for CLP vs Sham; ###P < 0.001, ####P < 0.0001 for NOX4^{tecKO}/CLP vs NOX4$^{fl/fl}$/CLP or for CLP + GKT vs CLP.

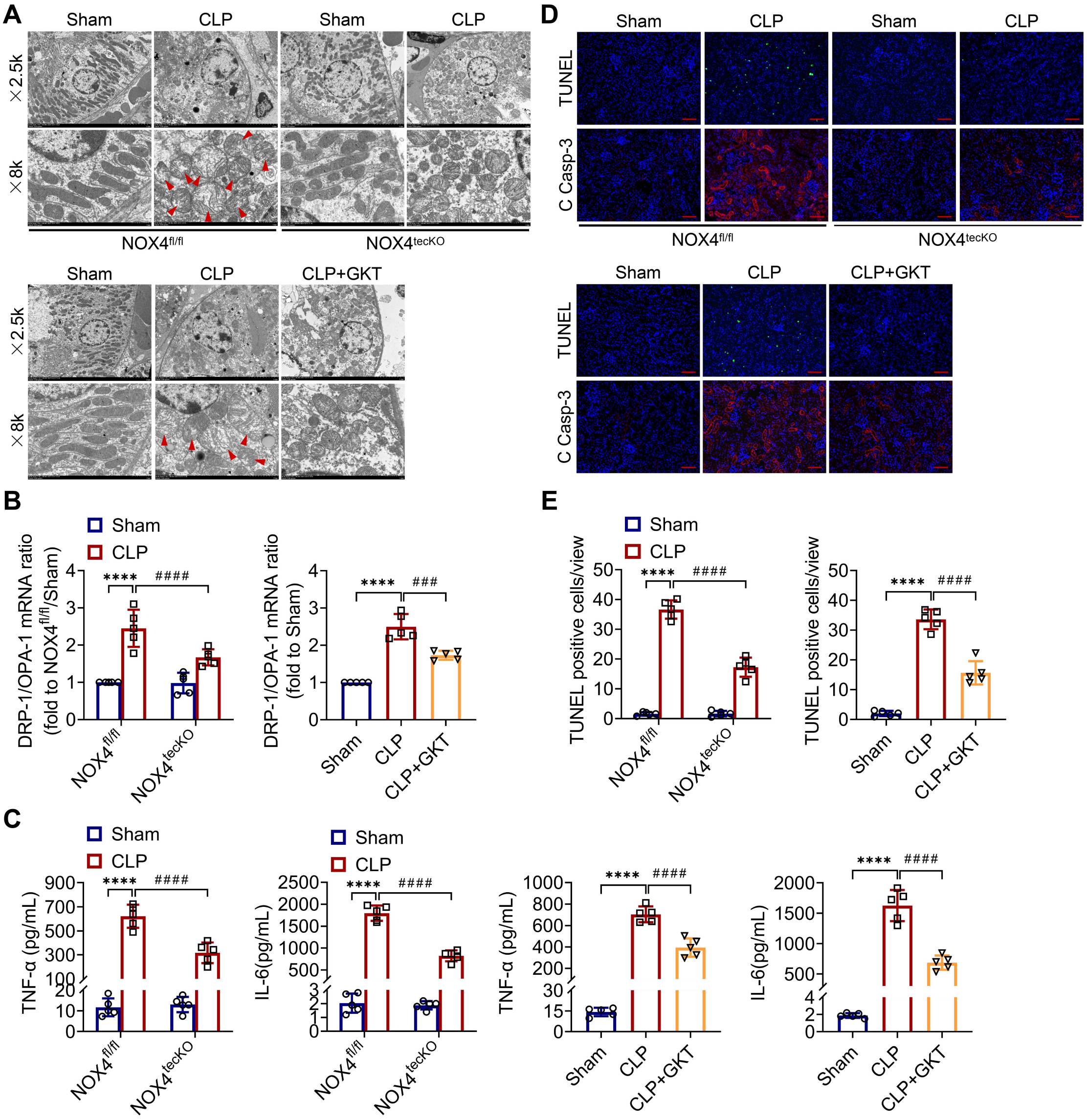

Figure S3. Genetic or pharmacologic inhibition of NOX4 alleviated CLP-induced kidney mitochondrial dysfunction, inflammation and cell apoptosis in mice. (A) Representative photomicrographs of mitochondria in RTECs collected by transmission electron microscopy (×2500, scale bars = 5 μm; ×8000, scale bars = 1 μm). The red triangle indicates injured mitochondria. (B) Gene expression ratio of DRP-1/OPA-1 in renal tissues measured by RT–qPCR. (C) Serum levels of TNF-α and IL-6 determined using ELISA kits. (D) Representative images of TUNEL staining and immunofluorescence staining of C Casp-3 (×200, scale bars = 50 μm). (E) Quantification of TUNEL-positive cells in the kidney cortex. Data are represented as

the mean ± SD, n = 5. $^{****}P < 0.0001$ for NOX4$^{fl/fl}$/CLP vs NOX4$^{fl/fl}$/Sham or for CLP vs Sham; $^{\#\#}P < 0.01$, $^{\#\#\#}P < 0.001$, $^{\#\#\#\#}P < 0.0001$ for NOX4^{tecKO}/CLP vs NOX4$^{fl/fl}$/CLP or for CLP + GKT vs CLP.

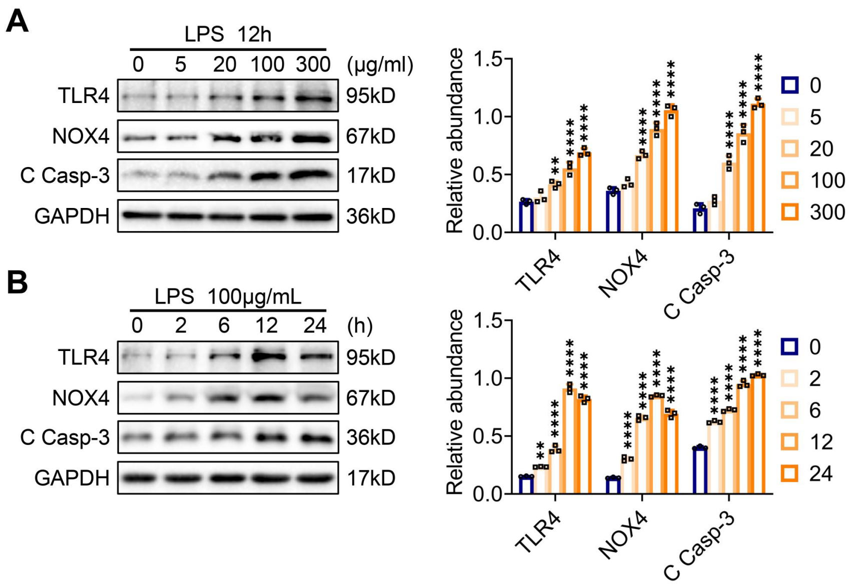

Figure S4. Effect of doses and timing of LPS stimulation in TCMK-1 cells.

TCMK-1 cells were treated with (A) 0-300 μg/mL LPS for (B) 0-24 h. Western blotting and densitometry quantification of TLR4, NOX4 and cleaved caspase-3 (C Casp-3) in TCMK-1 cells. All data are represented as mean ± SD (n = 3); $^{**}P < 0.01$, $^{****}P < 0.0001$ vs LPS (μg/mL) 0 group or LPS (h) 0 group.

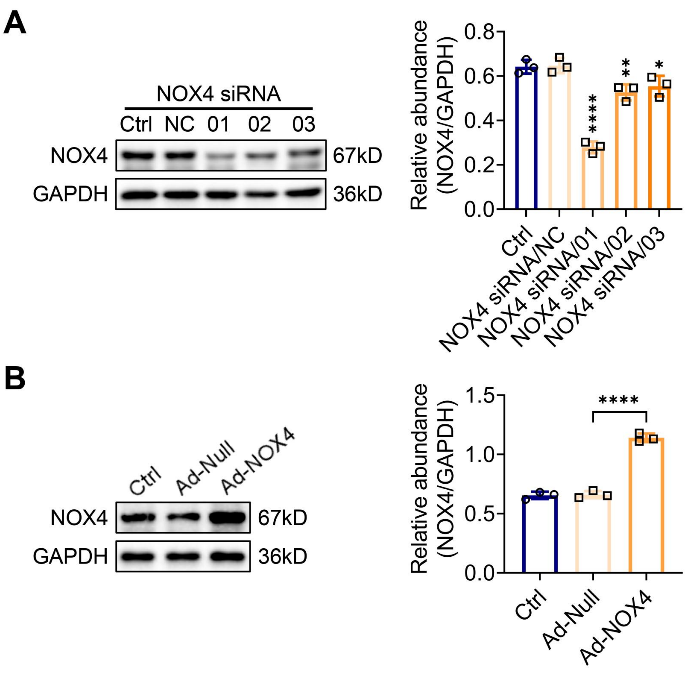

Figure S5. Western blot analysis of NOX4 knockdown by siRNA and over-expression by adenovirus. (A) TCMK-1 cells were transfected with NOX4 siRNA or negative control (NC) siRNA for 24 h to collect lysate for western blot analysis. (B) TCMK-1 cells were infected with adenovirus expressing NOX4 (Ad-NOX4) or empty Ad-Null for 24 h to collect lysate for western blot analysis. Data are shown as mean ± SD (n = 3); $^{*}P < 0.05$, $^{**}P < 0.01$, $^{****}P < 0.0001$ vs NOX4 siRNA/NC group or Ad-Null group.

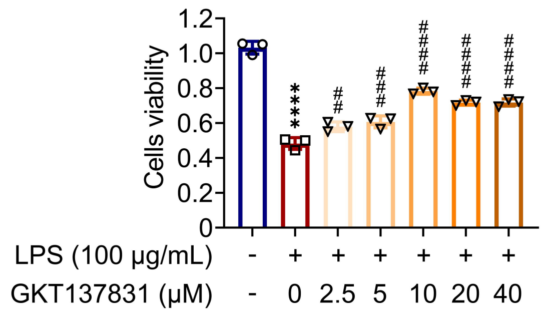

Figure S6. The cytoprotective effect of GKT137831 on LPS-stimulated TCMK-1 cells for 12 h. Cell viability was identified in this experiment relying on Cell Counting Kit-8 assay. Data are shown as mean ± SD (n = 3); ****P < 0.0001 vs GKT (μM) - with LPS (100 μg/mL) - group; $^{\#\#}$P < 0.01, $^{\#\#\#}$P < 0.001, $^{\#\#\#\#}$P < 0.0001 vs GKT (μM) 0 with LPS (100 μg/mL) + group.

3. Supplementary Tables

Table S1. Primary antibodies used in the experiments.

Name	Company	Catalog Number
Anti-NOX4	Abcam, MA, USA	ab133303
Anti-TNF-α	Affinity Biosciences, Changzhou, China	AF7014
Anti-IL-6	Zen BioScience, Chengdu, China	347023
Anti-MCP-1	R&D Systems, Minnesota, USA	AF-479-SP
Anti-BAX	Proteintech Group, Wuhan, China	50599-2-Ig
Anti-BCL-2	Proteintech Group, Wuhan, China	26593-1-AP
Anti-cleaved caspase-3	Cell Signaling Technology, MA, USA	9661
Anti-DRP-1	Novus Biologicals, Colorado, USA	NB110-55288
Anti-MFN-1	Proteintech Group, Wuhan, China	13798-1-AP
Anti-OPA-1	Novus Biologicals, Colorado, USA	NB110-55290
Anti-p-IκBα	Affinity Biosciences, Changzhou, China	AF2002

Anti-IκBα	Affinity Biosciences, Changzhou, China	AF5002
Anti-p-NF-κB p65	Cell Signaling Technology, MA, USA	3033
Anti-NF-κB p65	Santa Cruz Biotechnology, CA, USA	sc-8008
Anti-TLR4	Abcam, MA, USA	ab13556
Anti-GAPDH	Abcam, MA, USA	ab8245

Table S2. Sequences of the primers for quantitative real-time PCR.

Mouse Gene	Sequence
F-NGAL	GGAACGTTTCACCCGCTTTG
R-NGAL	CCACACTCACCACCCATTCA
F-TNF-α	ACCCTCACACTCAGATCATCTTC
R-TNF-α	TGGTGGTTTGCTACGACGT
F-IL-6	ACAACCACGGCCTTCCCTACTT
R-IL-6	CACGATTTCCCAGAGAACATGTG
F-MCP-1	TTAAAAACCTGGATCGGAACCAA
R-MCP-1	GCATTAGCTTCAGATTTACGGGT
F-Bax	TGAAGACAGGGGCCTTTTTG
R-Bax	AATTCGCCGGAGACACTCG
F-Bcl-2	GTCGCTACCGTCGTGACTTC
R-Bcl-2	CAGACATGCACCTACCCAGC
F-DRP-1	AGATGACCACCACTGTAGCC
R-DRP-1	AGCTTCCCCTTTCCCTGTTT
F-OPA-1	CTTCGTCTCTCCTCATCGGG
R-OPA-1	TGACATCCCACGCTGTACAG
F-GAPDH	AGGTCGGTGAACGGATTG
R-GAPDH	TGTAGACCATGTAGTTGAGGTCA
